# Supplementary material for: Assessing the extinction risk of the spontaneous flora in urban tree bases
Source: PLoS Comput Biol. 2024 Jun 27;20(6):e1012191. doi: 10.1371/journal.pcbi.1012191 (PMC11236206; doi:10.1371/journal.pcbi.1012191)
Supplement: S1 Text — Additional information regarding the Paris 12 dataset (including a map of the study area and a list of the species and streets taken into account as part of the study, see Tables A, B and Fig A) as well as the detailed results of the statistical tests performed. The list of these statistical tests can be found in the section “Material and Methods—Analysis of the Paris 12 dataset” of the manuscript, and a summary of these results in the “Results” section (subsections “Global extinction risk”, “Local extinction risk” and “Presence of a seed bank”). (PDF) [file pcbi.1012191.s001.pdf]

The Paris 12 dataset - Presentation and estimation results

Name of the street	Abbreviation	Number of tree bases
Rue Baron le Roy	BARO-1	40
	BARO-2	19
Boulevard de Bercy	BERC-1	22
	BERC-2	37
Rue de Charenton	CHAR-1	69
	CHAR-2	70
Rue Daumesnil	DAUM-1	102
	DAUM-2	44
	DAUM-3	39
Rue Joseph Kessel	KESS-1	36
	KESS-2	33
Rue Montgallet	MONT	48
Rue Pommard	POMM-1	17
	POMM-2	22
Quai de la Rapée	RAPE-1	49
	RAPE-2	25
Rue de Bercy	RBER-1	22
	RBER-2	18
Rue de Reuilly	REUI-1	36
	REUI-2	42
Rue Taine	TAIN-1	33
	TAIN-2	29

Table A: List of the portions of streets taken into account in this study

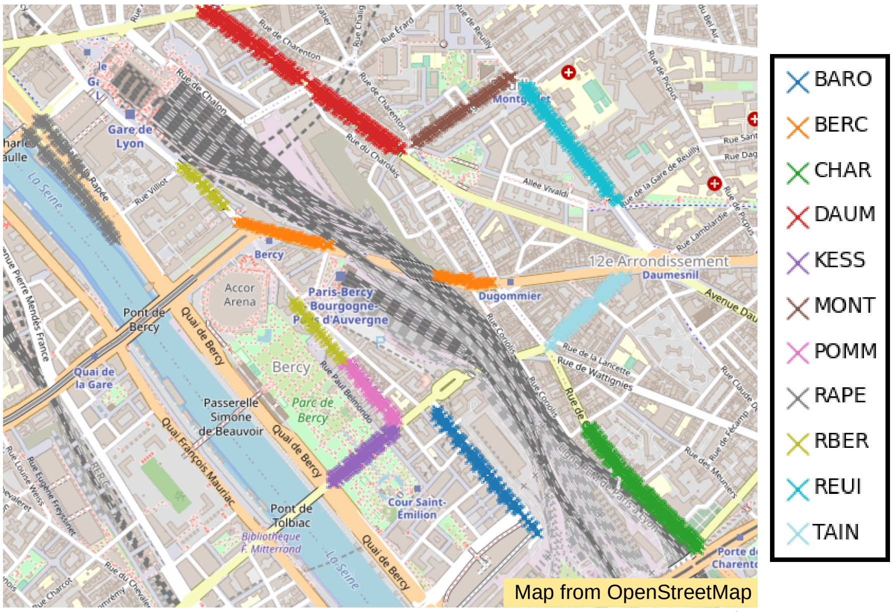

Figure A: Map of the study area. The full street names are listed in Table A. Map from OpenStreetMap (openstreetmap.org/copyright), adapted to add the locations of tree bases.

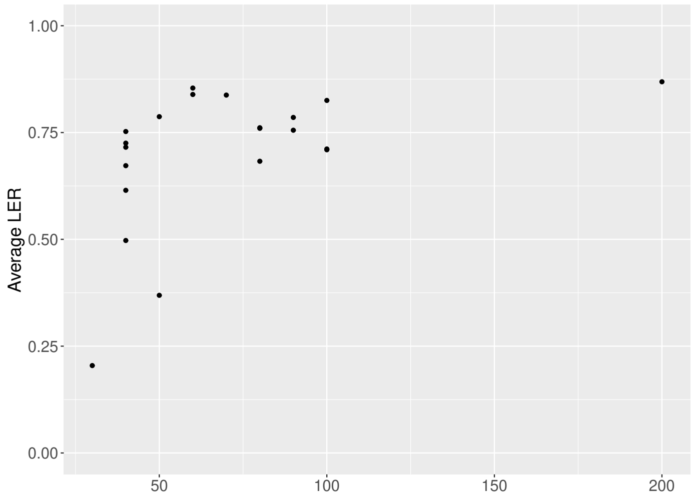

(a) Maximal height

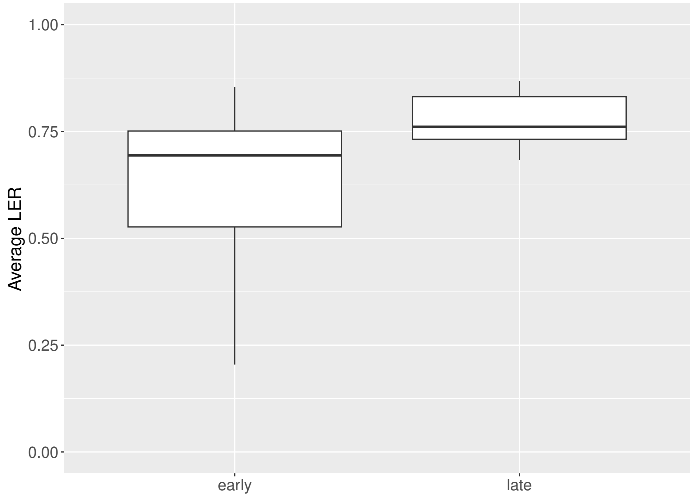

(b) Beginning of flowering period

Figure B: Illustration of the relation between the local extinction risk (as quantified by the LER metric, and averaged over all streets for each species) and two traits for which a borderline significant correlation was identified: (a) the maximal height (p-value = 0.01918), and (b) the beginning of the flowering period (p-value = 0.061). See Table 1 for more details on the plant traits and the correlation tests used.

Name of the plant species	Monitoring in 2013	Number of portions of streets	List of portions of streets
Bromus sterilis	No	2	BERC-1, KESS-2
Capsella bursa-pastoris	Yes	16	BARO-1, BARO-2, BERC-1, BERC-2, CHAR-1, CHAR-2, DAUM-1, DAUM-2, DAUM-3, KESS-1, KESS-2, POMM-1, POMM-2, RBER-1, RBER-2, REUI-1
Chenopodium album	Yes	4	BARO-1, BERC-1, RAPE-1, REUI-1
Conyza sp.	Yes	20	BARO-1, BARO-2, BERC-1, BERC-2, CHAR-1, DAUM-1, DAUM-2, DAUM-3, KESS-1, KESS-2, MONT, POMM-1, POMM-2, RAPE-1, RAPE-2, RBER-1, RBER-2, REUI-1, REUI-2, TAIN-1
Geranium molle	No	3	BARO-1, CHAR-1, CHAR-2
Hordeum murinum	Yes	16	BARO-1, BARO-2, BERC-1, BERC-2, CHAR-1, CHAR-2, DAUM-1, DAUM-2, DAUM-3, KESS-1, KESS-2, RAPE-1, RBER-1, RBER-2, REUI-1, REUI-2
Lactuca serriola	Yes	1	BARO-1
Lolium perenne	No	4	DAUM-1, DAUM-2, DAUM-3, KESS-2
Parietaria judaica	No	2	BARO-1, REUI-2
Plantago lanceolata	Yes	1	RAPE-1
Plantago major	Yes	5	BARO-1, POMM-2, RAPE-1, REUI-1, REUI-2
Poa annua	No	22	BARO-1, BARO-2, BERC-1, BERC-2, CHAR-1, CHAR-2, DAUM-1, DAUM-2, DAUM-3, KESS-1, KESS-2, MONT, POMM-1, POMM-2, RAPE-1, RAPE-2, RBER-1, RBER-2, REUI-1, REUI-2, TAIN-1, TAIN-2
Polygonum aviculare	Yes	11	BARO-1, BARO-2, BERC-1, BERC-2, CHAR-1, CHAR-2, DAUM-1, DAUM-3, RAPE-1, RAPE-2, REUI-1, REUI-2
Senecio inaequidens	Yes	2	BARO-1, BERC-2
Senecio vulgaris	Yes	6	BARO-2, KESS-1, POMM-1, RAPE-1, RBER-2, REUI-1
Sisymbrium irio	Yes	7	BARO-1, BARO-2, BERC-1, BERC-2, DAUM-1, KESS-1, MONT, REUI-1
Sisymbrium officinale	No	2	BARO-1, REUI-1
Sonchus oleraceus	No	13	BARO-1, BARO-2, BERC-1, BERC-2, DAUM-1, KESS-1, KESS-2, MONT, POMM-1, POMM-2, RAPE-1, RAPE-2, RBER-2
Stellaria media	Yes	22	BARO-1, BARO-2, BERC-1, BERC-2, CHAR-1, CHAR-2, DAUM-1, DAUM-2, DAUM-3, KESS-1, KESS-2, MONT, POMM-1, POMM-2, RAPE-1, RAPE-2, RBER-1, RBER-2, REUI-1, REUI-2, TAIN-1, TAIN-2
Taraxacum sp.	Yes	19	BARO-1, BARO-2, BERC-1, BERC-2, CHAR-1, CHAR-2, DAUM-1, DAUM-2, DAUM-3, KESS-1, KESS-2, MONT, POMM-1, RAPE-1, RAPE-2, RBER-1, RBER-2, REUI-1, REUI-2
Veronica persica	Yes	1	RAPE-1

Table B: List of the plant species taken into account in this study. The taxonomic reference used is the French Flora Reference TAXREF v8.0 (2014). For each species, the portion of streets which were taken into account in this study were the ones in which the focal species was observed in an average of at least 10% of the patches per year over the monitoring period (2009-2018 for species monitored in 2013, 2014-2018 otherwise).

	**Estimate**	**Standard error**	**p-value**
(Intercept)	0,127	0,161	0,4339
**Species**	**Estimate**	**Standard error**	**p-value**
Capsella bursa-pastoris	-0,111	0,158	0,4852
Chenopodium album	0,706	0,182	0,0002
Conyza sp.	-0,093	0,157	0,5525
Geranium molle	0,129	0,195	0,5087
Hordeum murinum	-0,118	0,158	0,4554
Lactuca serriola	0,841	0,259	0,0014
Lolium perenne	0,386	0,182	0,0353
Parietaria judaica	0,378	0,214	0,0790
Plantago lanceolata	1,003	0,261	0,0002
Plantago major	0,725	0,179	0,0001
Poa annua	-0,146	0,157	0,3521
Polygonum aviculare	0,587	0,162	0,0004
Senecio inaequidens	0,856	0,213	0,0001
Senecio vulgaris	0,054	0,174	0,7590
Sisymbrium irio	0,067	0,167	0,6893
Sisymbrium officinale	0,797	0,212	0,0003
Sonchus oleraceus	0,606	0,160	0,0002
Stellaria media	-0,146	0,157	0,3522
Taraxacum sp.	-0,145	0,157	0,3574
Veronica persica	0,074	0,261	0,7770
**Street**	**Estimate**	**Standard error**	**p-value**
BARO-2	0,129	0,086	0,1370
BERC-1	-0,110	0,084	0,1955
BERC-2	0,035	0,085	0,6781
CHAR-1	-0,027	0,092	0,7725
CHAR-2	0,080	0,096	0,4045
DAUM-1	0,002	0,086	0,9791
DAUM-2	0,056	0,097	0,5634
DAUM-3	0,039	0,093	0,6778
KESS-1	0,003	0,089	0,9738
KESS-2	0,038	0,091	0,6794
MONT	0,127	0,101	0,2132
POMM-1	0,060	0,097	0,5340
POMM-2	0,050	0,101	0,6232
RAPE-1	-0,129	0,086	0,1337
RAPE-2	0,053	0,101	0,6042
RBER-1	0,038	0,101	0,7112
RBER-2	-0,006	0,093	0,9509
REUI-1	-0,078	0,081	0,3354
REUI-2	0,049	0,091	0,5911
TAIN-1	0,002	0,132	0,9876
TAIN-2	0,020	0,157	0,9005

Table C: Summary of the results of the regression of MaxGER on species and portions of streets. For the species, estimates are expressed relative to *Bromus sterilis*. For the streets, estimates are expressed relative to BARO-1. The species in bold are the ones for which a significantly higher extinction risk was identified.

**Plant trait**	**p-value**
Dispersal mechanism	0.6899
**Flowering duration**	0.002677
Seed mass	0.5479
Heat preference	0.763
Pollination vector	0.5634
**Maximal height**	0.004763
**Beginning of flowering period**	0.00915

Table D: Summary of the results of the correlation tests of the global extinction risk (as quantified by the MaxGER metric and averaged over all streets for each species) with the species traits listed in Table 1. The traits in bold are the ones for which a significant correlation was identified (when accounting for multiple testing using the Holm-Bonferroni method).

	**Estimate**	**Standard error**	**p-value**
(Intercept)	0,745	0,091	0,0000
**Species**	**Estimate**	**Standard error**	**p-value**
Capsella bursa-pastoris	-0,053	0,090	0,5552
Chenopodium album	0,009	0,103	0,932
Conyza sp.	-0,010	0,089	0,9107
Geranium molle	0,022	0,111	0,8465
Hordeum murinum	-0,254	0,090	0,0054
Lactuca serriola	0,124	0,147	0,3993
Lolium perenne	0,078	0,103	0,4508
Parietaria judaica	0,012	0,121	0,9210
Plantago lanceolata	0,065	0,148	0,6599
Plantago major	0,109	0,102	0,2847
Poa annua	-0,578	0,089	0,0000
Polygonum aviculare	-0,071	0,092	0,4381
Senecio inaequidens	0,062	0,121	0,6096
Senecio vulgaris	0,111	0,099	0,2633
Sisymbrium irio	0,004	0,095	0,9625
Sisymbrium officinale	0,034	0,120	0,7797
Sonchus oleraceus	0,022	0,091	0,8103
Stellaria media	-0,110	0,089	0,2164
Taraxacum sp.	-0,267	0,089	0,0032
Veronica persica	-0,045	0,148	0,7602
**Street**	**Estimate**	**Standard error**	**p-value**
BARO-2	0,077	0,049	0,1182
BERC-1	-0,045	0,048	0,3518
BERC-2	0,046	0,048	0,3377
CHAR-1	-0,014	0,052	0,7915
CHAR-2	0,076	0,054	0,1635
DAUM-1	0,008	0,049	0,8677
DAUM-2	0,041	0,055	0,4634
DAUM-3	0,033	0,053	0,5351
KESS-1	-0,038	0,051	0,4494
KESS-2	-0,022	0,052	0,6724
MONT	0,082	0,057	0,1544
POMM-1	0,083	0,055	0,1326
POMM-2	0,066	0,057	0,2509
RAPE-1	-0,085	0,049	0,0833
RAPE-2	0,071	0,057	0,2197
**RBER-1**	0,118	0,057	0,0418
RBER-2	-0,001	0,053	0,9786
REUI-1	-0,046	0,046	0,3121
REUI-2	-0,009	0,052	0,8639
**TAIN-1**	0,163	0,075	0,0308
**TAIN-2**	0,229	0,089	0,0109

Table E: Summary of the results of the regression of LER on species and portions of streets. For the species, estimates are expressed relative to *Bromus sterilis*. For the streets, estimates are expressed relative to BARO-1. The species in grey are the ones for which a significantly lower LER was identified. The portions of streets in bold are the ones for which a significantly higher LER was identified.

Plant trait	p-value
Dispersal mechanism	0.2049
Flowering duration	0.2748
Seed mass	0.3375
Heat preference	0.814
Pollination vector	0.3371
Maximal height	0.01918
Beginning of flowering period	0.061

Table F: Summary of the results of the correlation tests of the local extinction risk (as quantified by the LER metric and averaged over all streets for each species) with the species traits listed in Table 1. No significant correlation was identified when accounting for multiple testing (using the Holm-Bonferroni method).

Value of H_{inf}	Species
$H_{inf} = 0$	***Plantago lanceolata***<br>*Sisymbrium officinale* (*)
$H_{inf} = 1$	*Chenopodium album*<br>*Polygonum aviculare*<br>*Senecio inaequidens*<br>*Veronica persica*
$H_{inf} = 2$	*Parietaria judaica*<br>*Sonchus oleraceus*
$H_{inf} = 3$	*Plantago major*<br>*Poa annua*
$H_{inf} = 4$	*Lactuca serriola*<br>*Sisymbrium irio*<br>*Bromus sterilis*<br>*Geranium molle*<br>*Lolium perenne*
$H_{inf} = 5$	\
$H_{inf} = 6$	\
$H_{inf} = 7$	*Conyza sp.*<br>*Hordeum murinum*<br>*Senecio vulgaris*<br>*Taraxacum sp.*
$H_{inf} = 8$	*Capsella bursa-pastoris*<br>*Stellaria media*

Table G: Value of $H_{inf} = \min\{h \in [\![0, H_{max}]\!] : \mathbb{P}(H \leq h|\text{Obs}) \geq 0.05\}$ for each species listed in Table B. The posterior distribution of H was obtained by performing parameter inference simultaneously on all portions of streets listed in Table B, assuming that only p_{ext} and s differed from one portion of street to another. Species in bold are species for which the absence of a seed bank was identified (that is, for which $\mathbb{P}(H = 0|\text{Obs}) \geq 0.95$). The asterisk indicates species for which neither the absence (see above) nor the presence (defined as $\mathbb{P}(H \geq 1|\text{Obs}) \geq 0.95$) of a seed bank was identified.

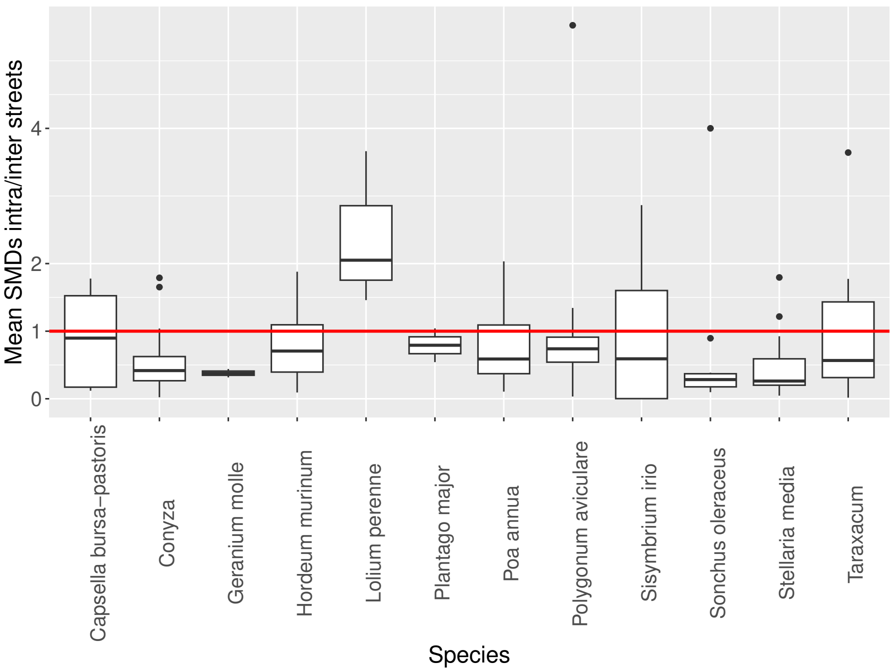

(a) Distribution of the quotients of the average SMDs inside a street and between streets, grouped by species.

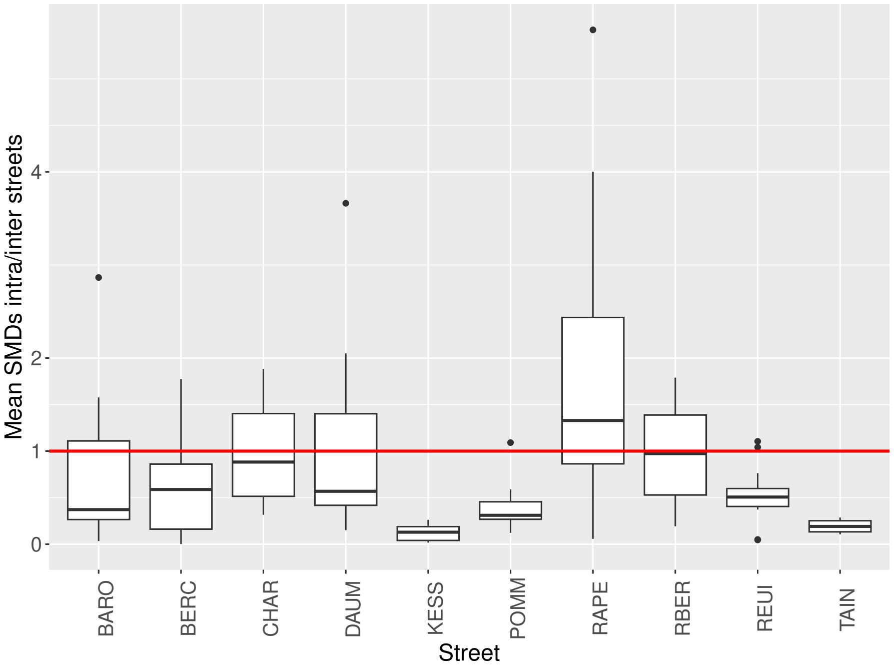

(b) Distribution of the quotients of the average SMDs inside a street and between streets, grouped by street.

Figure C: Comparison of the average *Standardised Mean Differences* (SMDs) of the posterior distributions of patch extinction probabilities computed between portions of the same streets or of different streets. The plots correspond to the distribution of the quotients of the mean SMDs inside a street and between streets, grouped by species (a) or by street (b).
We recall that the SMD measures the difference between two probability distributions. Therefore, a quotient smaller than one indicates that posterior distributions of patch extinction probabilities are on average closer between portions of a same street than between portions of different streets, while a quotient larger than one indicates the opposite.
