# Supplementary material for: Assessing the extinction risk of the spontaneous flora in urban tree bases
Source: PLoS Comput Biol. 2024 Jun 27;20(6):e1012191. doi: 10.1371/journal.pcbi.1012191 (PMC11236206; doi:10.1371/journal.pcbi.1012191)
Supplement: S2 Text — Presentation of the framework implemented to perform inference under a BOA process. The corresponding code can be found online at https://github.com/cmantoux/boa-process, along with detailed explanations regarding how to reproduce the results presented in this article. (PDF) [file pcbi.1012191.s002.pdf]

The BOA Process: Mathematical formulation and estimation procedure

1 Formal definition of the BOA process

Formally, the BOA process for a street with N patches observed for T years involves three dependent random variables:

1. The *seed age* $L = (L_{n,t})_{n\in[\![1,N]\!],t\in[\![1,T]\!]} \in \mathbb{N}^{N\times T}$.

   The random variable $L_{n,t}$ gives the age of the youngest seeds in patch n at the beginning of generation t. These seeds are *viable* (i.e., they can germinate and grow into plants) if and only if their age is below the *maximal dormancy duration* H.

2. The *extinction events* $E = (E_{n,t})_{n\in[\![1,N]\!],t\in[\![1,T]\!]} \in \{0,1\}^{N\times T}$.

   We have $E_{n,t} = 1$ if an extinction event occurs in patch n during generation t, and $E_{n,t} = 0$ otherwise.

3. The *observations of standing vegetation* $O = (O_{n,t})_{n\in[\![1,N]\!],t\in[\![1,T]\!]} \in \{0,1\}^{N\times T}$.

   We have $O_{n,t} = 1$ if plants are observed in patch n during generation t, after potential patch extinction events and before the seed production step, and we have $O_{n,t} = 0$ otherwise.

At $t = 1$ (initial condition), each patch is randomly chosen to contain viable seeds with probability $s \in [0,1]$. In that case, the age of the youngest viable seeds is chosen uniformly at random in $\{0,\dots,H\}$. Then, during each generation t, we follow these five steps.

1. Germination step: In all patches n such that $L_{n,t} \le H$ (i.e., in all patches containing viable seeds), some of the viable seeds germinate and grow into plants.

2. Extinction step: Each patch containing plants is affected by an extinction event with probability $p_{ext} \in [0,1]$, independently of other patches. This extinction event kills all the plants in the patch. Formally, the random variables $(E_{n,t})_{1\le n\le N}$ are i.i.d. and follow a Bernoulli distribution with parameter $p_{ext} \in [0,1]$. The patch n is affected by an extinction event during generation t if, and only if $E_{n,t} = 1$.

3. Observation step: The observer records which patches contain standing vegetation. For each patch n, $O_{n,t}$ is set to 1 if the patch contain plants, and to 0 otherwise.

4. Seed production step: For each patch such that $O_{n,t} = 1$, we set

$$L_{n,t+1} = L_{\max(1,n-1),t+1} = L_{\min(N,n+1),t+1} = 0.$$

For all remaining non-affected patches n', we set $L_{n',t+1} = L_{n',t} + 1$.

5. All remaining plants die, for instance due to the action of gardeners.

The BOA process is thus characterised by three parameters:

- The initial *proportion of occupied patches* $s \in [0, 1]$,
- The *maximal dormancy duration* $H \in \mathbb{N}$,
- The *patch extinction probability* $p_{\text{ext}} \in [0, 1]$.

2 Bayesian framework used

2.1 Model likelihood

With the above description of the BOA process, we can now write the probability $\mathbb{P}(O, L \mid s, p_{\text{ext}}, H)$ that the observations O and the seed ages L are produced by the process, given that the true parameters are s, p_{ext} and H. Let us introduce the shortcut notations $O_t = (O_{1,t}, ..., O_{N,t})$ and $L_t = (L_{1,t}, ..., L_{N,t})$.

As a first remark, note that the age L_t of the seeds at times $t > 1$ is a deterministic function of the initial age of the seeds L_1 and the previous observations $O_1, ..., O_{t-1}$. This function defines the seed production process; it can be written recursively as:

$$L_{n,t+1} = \begin{cases} L_{n,t} + 1 & \text{if } O_{\min(1,n-1),t} = O_{n,t} = O_{\max(N,n+1),t} = 0 \\ 0 & \text{otherwise.} \end{cases}$$

As a consequence, we only need to compute the probability $\mathbb{P}(O, L_1 \mid s, p_{\text{ext}}, H)$, since the remaining values $L_2, ..., L_T$ are a deterministic function of L_1 and O. This probability can be decomposed as:

$$\mathbb{P}(O, L_1 \mid s, p_{\text{ext}}, H) = \mathbb{P}(L_1 \mid s, p_{\text{ext}}, H) \times \mathbb{P}(O \mid L_1, s, p_{\text{ext}}, H).$$

At time $t = 1$, under the definition of the process introduced in the previous section, the seeds L_1 are either non viable (which we represent with a seed age equal to $H + 1$), or viable, with an age chosen uniformly in $\{0, \ldots, H\}$. Therefore, for all $1 \leq n \leq N$, $L_{n,1} \in \{0, ..., H + 1\}$ and

$$\mathbb{P}(L_{n,1} = \ell \mid s, p_{\text{ext}}, H) = \begin{cases} \frac{s}{H+1} & \text{if } \ell \leq H \\ 1 - s & \text{if } \ell = H + 1. \end{cases}$$

As $L_{1,1}, ...L_{N,1}$ are independent, we can then compute $\mathbb{P}(L_1 \mid s, p_{\text{ext}}, H)$ as

$$\mathbb{P}(L_1 \mid s, p_{\text{ext}}, H) = \mathbb{P}(L_{1,1} \mid s, p_{\text{ext}}, H) \times \cdots \times \mathbb{P}(L_{N,1} \mid s, p_{\text{ext}}, H).$$

The probability $\mathbb{P}(O \mid L_1, s, p_{\text{ext}}, H)$ can be decomposed under the form

$$\mathbb{P}(O \mid L_1, s, p_{\text{ext}}, H) = \prod_{t=1}^{T} \mathbb{P}(O_t \mid L_t, s, p_{\text{ext}}, H).$$

This equation expresses the fact that the random variables O_t only depends on the quantity L_t (which is itself a function of $O_1, ..., O_{t-1}$, as described above). Then, each term in this equation can be expressed separately, as follows:

$$\mathbb{P}(O_{n,t} = 1 \mid L_t, s, p_{\text{ext}}, H) = \begin{cases} 1 - p_{\text{ext}} & \text{if } L_{n,t} \leq H \\ 0 & \text{otherwise.} \end{cases}$$

This equation expresses the fact that plants can only be observed in patch n during generation t if 1) $L_t \leq H$ (i.e., if the youngest seeds present in the patch are viable) and 2) if no extinction event occurred (which happens with probability $1 - p_{\text{ext}}$). As $O_{n,t}$ is $\{0, 1\}$-valued, we also obtain $\mathbb{P}(O_{n,t} = 0 \mid L_t, s, p_{\text{ext}}, H)$ as $1 - \mathbb{P}(O_{n,t} = 1 \mid L_t, s, p_{\text{ext}}, H)$.

A consequence of the above observation is that conditionally on L_t, the random variables $O_{1,t}, ..., O_{N,t}$ are independent. Therefore, we can compute $\mathbb{P}(O_t \mid L_t, s, p_{\text{ext}}, H)$ as

$$\mathbb{P}(O_t \mid L_t, s, p_{\text{ext}}, H) = \mathbb{P}(O_{1,t} \mid L_t, s, p_{\text{ext}}, H) \times \cdots \times \mathbb{P}(O_{N,t} \mid s, p_{\text{ext}}, H).$$

We can combine these expressions to obtain the complete likelihood of the model, which is given by $\mathbb{P}(O, L_1 \mid s, p_{\text{ext}}, H)$. Note that this quantity is different from the marginal likelihood of the observations $\mathbb{P}(O \mid s, p_{\text{ext}}, H)$. The latter would be ideally the one to use in practice (since L_1 is unknown), but is unfortunately impossible to compute: formally, it writes as sum over all the $(H+2)^N$ possible configurations of seed ages at time $t = 1$, and the number of such configurations grows exponentially with N. As a consequence, we need to estimate L_1 along with the model parameters s, p_{ext}, H.

2.2 Bayesian methodology

Since the observed data only spans a period of ten years, our estimation necessarily comprises a certain amount of uncertainty. In the context of complex hierarchical statistical models (of which the model

considered here is an example), Maximum Likelihood estimations only give the most likely value for s, p_{ext} and H, and cannot be used to obtain confidence regions around the estimated values. For this reason, methods like the EM are not suited to our study.

Instead, we propose to work in a Bayesian framework. It consists in determining the posterior distribution $\mathbb{P}(s, p_{\text{ext}}, H \mid O)$ of the parameters s, p_{ext}, H given the observed data O using Bayes' rule:

$$\mathbb{P}(s, p_{\text{ext}}, H \mid O) = \frac{\mathbb{P}(O \mid s, p_{\text{ext}}, H)\mathbb{P}(s, p_{\text{ext}}, H)}{\mathbb{P}(O)} .$$

As we will see in the following sections, this formula can be used to draw samples from the distribution $\mathbb{P}(s, p_{\text{ext}}, H \mid O)$, even though it may not be computed explicitly in practice. These samples are then used to approximate the distribution. For instance, if we have M samples $(s^{(m)}, p_{\text{ext}}^{(m)}, H^{(m)})_{1 \le m \le M}$ of the posterior distribution, the expectation of p_{ext} given O can be computed with a Monte-Carlo approximation:

$$\mathbb{E}[p_{\text{ext}} \mid O] \simeq \bar{p}_{\text{ext}} = \frac{1}{M} \sum_{m=1}^{M} p_{\text{ext}}^{(m)} .$$

Similarly, the uncertainty on p_{ext} can be measured by computing its posterior *variance*

$$\text{Var}(p_{\text{ext}} \mid O) \simeq \frac{1}{M} \sum_{m=1}^{M} \left(p_{\text{ext}}^{(m)} - \bar{p}_{\text{ext}}\right)^2 .$$

Prior distribution In a Bayesian framework, the model parameters s, p_{ext} and H are considered as random variables. In order to apply Bayes' rule and obtain the posterior distribution that we are interested in, their distribution *a priori* $\mathbb{P}(s, p_{\text{ext}}, H)$ must be defined in a way that reflects our (prior) knowledge of the model parameters. In this paper, we do not make any initial assumption on the model parameters, and consider the following simple, uninformative prior distributions:

- The initial proportion of occupied patches s follows a uniform distribution over $[0, 1]$.
- Similarly, p_{ext} also follows a uniform distribution over $[0, 1]$.
- The maximal dormancy duration H follows a uniform distribution over the set of integers $\{0, \ldots, H_{\max}\}$:

$$\mathbb{P}(H) = \begin{cases} 1/(H_{\max} + 1) & \text{if } H \in \{0, \ldots, H_{\max}\} \\ 0 & \text{otherwise.} \end{cases}$$

The pre-defined upper bound on the maximal dormancy duration is a consequence of the fact that with a limited number of years of observation, identifying long maximal dormancy durations (compared to the length of the observation window) can be an ill-posed problem and lead to identifiability issues.

We can then combine the different priors to obtain the complete prior distribution.

2.3 Markov Chain Monte-Carlo sampler

In order to compute the GER and the MaxGER extinction metrics, we need samples from the posterior distribution $\mathbb{P}(p_{\text{ext}}, H \mid O)$. As explained earlier, this distribution takes an intractable form: using the law of total probabilities it writes as

$$\mathbb{P}(p_{\text{ext}}, H \mid O) = \sum_{L_1 \in \{0,\dots,H+1\}^N} \int_0^1 \frac{\mathbb{P}(O, L_1, s, p_{\text{ext}}, H)}{\mathbb{P}(O)} ds\,,$$

which is a sum over an exponential number of terms. We overcome this hurdle by focusing instead on the posterior distribution of $(L_1, s, p_{\text{ext}}, H)$ given O, which writes as:

$$\mathbb{P}(L_1, s, p_{\text{ext}}, H \mid O) = \frac{\mathbb{P}(O, L_1, s, p_{\text{ext}}, H)}{\mathbb{P}(O)}.$$

Since this probability distribution is known up to a normalising constant, the method presented in the next section can be used to draw samples from it. This method will provide us with samples $(L_1^{(m)}, s^{(m)}, p_{\text{ext}}^{(m)}, H^{(m)})_{1\leq m\leq M}$, which then give the marginal distribution samples $(p_{\text{ext}}^{(m)}, H^{(m)})_{1\leq m\leq M}$ of the posterior distribution $\mathbb{P}(p_{\text{ext}}, H \mid O)$ that we are interested in.

In practice, we will use a Markov Chain Monte Carlo (MCMC) sampler to draw approximate samples from the posterior distribution of $\mathbb{P}(L_1, s, p_{\text{ext}}, H \mid O)$. Such methods rely on simple and fast iterations which generate a Markov chain whose invariant distribution is the target distribution we wish to sample from. After a certain amount of iterations, the samples of the Markov chain are very close from being true samples of the target distribution; they can thus be used in Monte-Carlo estimators to compute the GER and MaxGER metrics. Note that consecutive MCMC samples are correlated by definition, which increases the variance of Monte-Carlo estimations compared with independent samples, but does not affect the expectation of the estimator, which is unbiased as the number of samples grows large.

2.4 Metropolis-Hastings within Gibbs sampler

In this section, we detail the MCMC implemented in practice. For an introduction to classical MCMC methods, we refer the reader to (Robert and Casella, 2010). In this paper, we use the Metropolis-Hastings within Gibbs (MHwG) sampling algorithm, which is designed to draw samples from a general probability distribution $\pi(x_1, ..., x_d)$ in a high-dimensional space. The general MHwG procedure is recalled in Algorithm 1. In the algorithm, x_{-k} denotes the vector x where component k has been

removed: the term $\pi(x_k \mid x_{-k})$ thus refers to the conditional distribution of x_k given the remaining components of x. The algorithm mainly relies on the so-called proposal distributions q_k^m: at each step m, the distribution q_k^m proposes, given the m-th value of x_{-k}, a random candidate for the next value of x_k. Then, this candidate is accepted as the next value of x_k with probability α.

Algorithm 1: The Metropolis-Hastings within Gibbs algorithm

```
input : Target distribution π(x_1, ..., x_d); number of samples M
Initialize x_1^0, ..., x_d^0
for m = 1 to M do
    for k = 1 to d do
        Sample a candidate variable y_k^m from a proposal distribution q_k^m.
        Define the acceptance ratio α = min[1, q_k^m(y_k^m) π(x_k^{m-1} | x_{-k}^{m-1}) / (q_k^m(x_k^{m-1}) π(y_k^m | y_{-k}^m))]
        Sample B_k^m ~ Bernoulli(α).
        If B_k^m = 0, set x_k^m = x_k^{m-1}. Otherwise, set x_k^m = y_k^m.
    end
end
return (x^m)_{1≤m≤M}
```

MHwG for the BOA process In this paper, we are interested in the distribution $\mathbb{P}(L_1, s, p_{\text{ext}}, H \mid O)$. In the MHwG algorithm, we will thus alternatively be sampling from s, p_{ext}, H and the components of L_1. Since L_1 has N components, at each step of the algorithm we only update a single coordinate $L_{n,1}$ for a patch n chosen at random. This allows spending more time on sampling the model parameters s, p_{ext}, H rather than the seed ages $L_{n,1}$, which in practice only contribute marginally to the likelihood. In order to obtain a complete algorithmic procedure, we need to specify the proposal distributions q_k^m used at each step.

- For H: the new value of H is chosen uniformly at random in $\{0, ..., H_{\max}\}$.
- For $L_{n,1}$: the random value is sampled uniformly in $\{0, \ldots, H+1\}$. Values between 0 and H produce viable seeds, and the value $H+1$ produces non-viable seeds.
- For p_{ext} and s: instead of working with p_{ext} and s, we represent them as $p_{\text{ext}} = h(\xi_p)$ and $s = h(\xi_s)$, with $h(x) = (1+\exp(-x))^{-1} \in]0, 1[$ a sigmoid function. In the algorithm, the proposal value for ξ_p at step m is drawn from a Gaussian distribution $\mathcal{N}(\xi_p^{m-1}, \sigma_p^2)$. Similarly, the proposal value for ξ_s is drawn from a Gaussian distribution $\mathcal{N}(\xi_s^{m-1}, \sigma_s^2)$. The corresponding values for p_{exp} and s are then obtained by applying h. The sigmoid representation allows sampling values very close to 0 or 1 as well as more centered values using a common proposal variance σ_p^2 or σ_s^2.

In the form we just described, the algorithm currently has a practical issue: a change in the value of H can change the initial state of some seeds from viable to non-viable, and conversely. Although

this is not a problem in itself, we noticed that it hinders the convergence in practice. Our intuition is that it is due to the fact that frequent changes in the value of H prevent the distribution of L_1 from converging, as the viability of the seeds varies not only with the value of $L_{n,1}$, but also with H.

We overcome this issue by defining a duplicate L_1^h of L_1 for each value of $h \in \{0, \dots, H_{\max}\}$. For each h, we impose that $L_1^h \in \{0, h+1\}$; the distribution of L_1 given $H = h$ corresponds to L_1^h. In other words, by definition:

$$L_1 = \sum_{h=1}^{H_{\max}} \mathbb{1}_{\{H=h\}} L_1^h \, .$$

In practice, at each step of the MHwG algorithm, a random patch n is selected, and the variables $L_{n,1}^0, \dots, L_{n,1}^{H_{\max}}$ are updated. Theoretically, these variables should be updated using the conditional distribution $\mathbb{P}(L_{n,1}^h \mid s = s^{(m)}, p_{\text{ext}} = p_{\text{ext}}^{(m)}, H = H^{(m)}, O)$. However, it must be noted that, if $h \neq H^{(m)}$, the value of L_1^h does not play a role in the distribution of O. In other words, the variables L_1^h and O are independent conditionally on $H = H^{(m)}$, and the conditional distribution of L_1^h thus simplifies to a fixed base distribution over $\{0, \dots, h+1\}$. As a consequence, if $h \neq H^{(m)}$, the variable L_1^h is randomised to a distribution that does not depend on O. In order to bypass this hurdle, we change the conditional distribution $\mathbb{P}(L_{n,1}^h \mid s = s^{(m)}, p_{\text{ext}} = p_{\text{ext}}^{(m)}, H = H^{(m)}, O)$ in the MHwG to $\mathbb{P}(L_{n,1}^h \mid s = s^{(m)}, p_{\text{ext}} = p_{\text{ext}}^{(m)}, H = h, O)$. This modification results in an approximate MHwG procedure, which provides significantly better performances than the base algorithm.

As a last point, the multiple variables $L_1^0, \dots, L_1^{H_{\max}}$ must be modelled by distinct values of s: the proportion of viable seeds may vary depending on the value of h. In practice, s is thus replaced with a vector $(s^0, \dots, s^{H_{\max}}) \in [0,1]^{H_{\max}+1}$, and the probability distribution of L_1^h is defined using s^h.

Remark 0.1. The variances σ_p^2 and σ_s^2 for the proposal distributions of ξ_p and ξ_s are tuned throughout the convergence of the MCMC in order to obtain an average proportion of accepted samples around 30%. Larger variances lead to larger transitions, which are thus rejected more often; smaller variances lead to smaller transitions, which are easier to accept.

3 The noisy BOA process

The noisy BOA process adds a new parameter $\varepsilon \in [0,1]$, which controls the probability of what can be interpreted as external colonisation (but was introduced to model noise in observations). Compared to the standard BOA process, patches containing non-viable seeds or affected by an extinction event can now contain plants with probability ε. This only changes the probability $\mathbb{P}(O_{n,t} = 1 \mid L_t, s, p_{\text{ext}}, H, \varepsilon)$:

it now expresses as

$$\mathbb{P}(O_{n,t} = 1 \mid L_t, s, p_{\text{ext}}, H, \varepsilon) = \begin{cases} 1 - p_{\text{ext}}(1-\varepsilon) & \text{if } L_{n,t} \leq H \\ \varepsilon & \text{otherwise.} \end{cases}$$

As with s, p_{ext} and H, the posterior distribution of the parameter ε can be sampled from with the MHwG algorithm. As the parameter ε was introduced to provide a buffer against noise in the dataset rather than to model an actual biological phenomenon, we expect it to take very small values. Therefore, we take a *slack and slab* prior distribution for ε, that is to say, a mixture of two uniform distributions:

$$\varepsilon \sim \frac{1}{2}\mathcal{U}([0, \varepsilon_m]) + \frac{1}{2}\mathcal{U}([0, \varepsilon_M]).$$

In practice, we choose $\varepsilon_m \ll \varepsilon_M$, e.g., $\varepsilon_m = 1\%$ and $\varepsilon_M = 5\%$. The idea is to obtain a prior distribution concentrated around relatively small values, with a spike on very small values.

We perform Metropolis transitions on the inverse sigmoid of ε, $\xi_\varepsilon = h^{-1}(\varepsilon)$, with a Gaussian proposal with variance a σ_ε^2 tuned adaptively throughout the MCMC convergence.

4 Handling multiple streets

Finally, the method introduced in the previous sections seamlessly transposes to the case of observations of a given species in multiple streets, denoted $O_1, ..., O_K$. We assume that for each species, the maximum dormancy duration H and the noise intensity ε are identical across all streets, but the initial proportion of occupied patches s_k and the extinction probability $(p_{\text{ext}})_k$ depend the street $1 \leq k \leq K$. As in the previous case, we are interested in sampling from the distribution

$$\mathbb{P}((p_{\text{ext}})_1, \ldots, (p_{\text{ext}})_K, H, \varepsilon \mid O_1, \ldots, O_K),$$

and, as in the previous case, we tackle this problem by instead sampling from the complete posterior distribution

$$\mathbb{P}((L_1, s, p_{\text{ext}})_1, \ldots, (L_1, s, p_{\text{ext}})_K, H, \varepsilon \mid O_1, \ldots, O_K),$$

which, up to the normalising constant $\mathbb{P}(O_1, \ldots, O_K)$, is proportional to the function

$$\mathbb{P}(O_1, \ldots, O_K, (L_1, s, p_{\text{ext}})_1, \ldots, (L_1, s, p_{\text{ext}})_K, H, \varepsilon).$$

The MHwG can then be transposed to the multiple streets setting, by updating H, ε and the parameters $(L_1, s, p_{\text{ext}})_k$ of each street k at each MHwG step.

Using multiple streets at the same time allows reducing the uncertainty on the parameters H and ε, which benefit from the combination of all the observations; this is verified in our numerical experiments (see S3 Text, Section 4).

References

Robert, C., & Casella, G. (2010, November). *Monte Carlo Statistical Methods.* Springer.
