# Supplementary material for: Assessing the extinction risk of the spontaneous flora in urban tree bases
Source: PLoS Comput Biol. 2024 Jun 27;20(6):e1012191. doi: 10.1371/journal.pcbi.1012191 (PMC11236206; doi:10.1371/journal.pcbi.1012191)
Supplement: S3 Text — Detailed results of the assessment of the performance of the MaxGER and GER metrics, as described in the section “Material and Methods—Measuring the extinction risk” of the manuscript. (PDF) [file pcbi.1012191.s003.pdf]

Assessment of the performances of the estimation procedure

1 Parameter sets

Parameter	Values
Number of patches N	50, 100
Number of years of observation T	5, 10
Initial proportion of occupied patches s	0.2, 0.8
Patch extinction probability p_{ext}	0.1, 0.2, ..., 0.9
Maximal dormancy duration H	0, 1, 2, 5
Noise intensity ϵ	0.00, 0.01, 0.02, 0.05
Additional parameter sets - $H = 0$	
Patch extinction probability p_{ext}	$p_c(0) + x$, $x = -0.1$, -0.08, ..., 0.08, 0.1
Additional parameter sets - $H = 1$	
Patch extinction probability p_{ext}	$p_c(1) + x$, $x = -0.1$, -0.08, ..., 0.08, 0.1
Additional parameter sets - $H = 2$	
Patch extinction probability p_{ext}	$p_c(2) + x$, $x = -0.1$, -0.08, ..., 0.08, 0.1
Additional parameter sets - $H = 5$	
Patch extinction probability p_{ext}	$p_c(5) + x$, $x = -0.1$, -0.08, ..., 0.08, 0.1

Table A: Parameter sets used to compare the performances of the MaxGER and GER metrics. For each parameter set, we simulated 30 BOA processes, and computed the average MaxGER and GER over the 30 simulated processes.

Parameter	Values
Number of patches N	50
Number of years of observation T	10
Initial proportion of occupied patches s	0.2, 0.8
Patch extinction probability p_{ext}	0.1, 0.35, 0.55, 0.75
Maximal dormancy duration H	0, 1, 2, 5
Additional parameter - False positives	
False positive rate ϵ_{pos}	0.00, 0.01, 0.02, 0.05
Additional parameter - False negatives	
False negative rate ϵ_{neg}	0.00, 0.01, 0.02, 0.05
Additional parameter - External colonization	
External colonization rate ϵ_{col}	0.00, 0.01, 0.02, 0.05

Table B: Parameter sets used to compare the performances of the MaxGER and GER metrics on corrupted datasets (containing false positives, false negatives or external colonization). For each parameter set, we simulated 30 BOA processes and computed the average MaxGER and GER over the 30 simulated processes.

Parameter	Values
Number of patches N	30, 50
Number of years of observation T	5, 10
Initial proportion of occupied patches s	0.2, 0.8
Patch extinction probability p_{ext}	0.1, 0.35, 0.55, 0.75
Maximal dormancy duration H	0, 1, 2, 5
Noise intensity ϵ	0, 0.01, 0.02, 0.05
Number of streets M	1, 2, 5, 10

Table C: Parameter sets used to assess the effect of performing the estimation simultaneously on multiple streets on the quality of the estimation of H. For each parameter set, we generated 30 simulations and performed parameter inference assuming that H and ϵ were constant across streets.

2 Comparison of the performances of the GER and MaxGER metrics

2.1 Performances of the GER metric

The following figures show the evolution of the GER metric as a function of the patch extinction probability p_{ext}. For each parameter set listed in Table A, we simulated 30 datasets and performed parameter inference under a noisy BOA process. We then computed the average GER across the 30 simulations. The black vertical line indicates the critical patch extinction probability $p_c(H)$.

$$(N, T) = (50, 5)$$

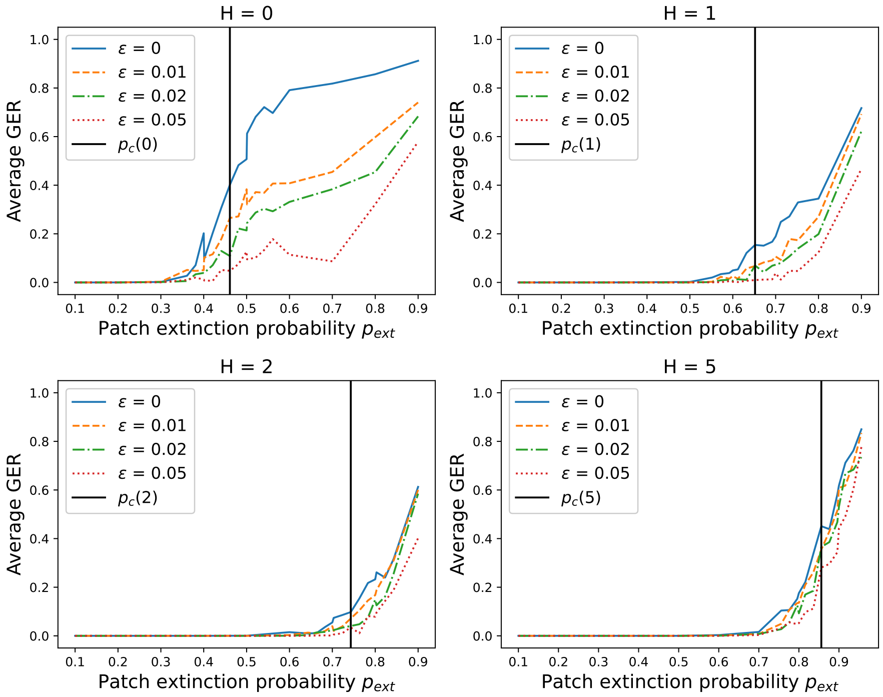

Figure A

$(N, T) = (50, 10)$

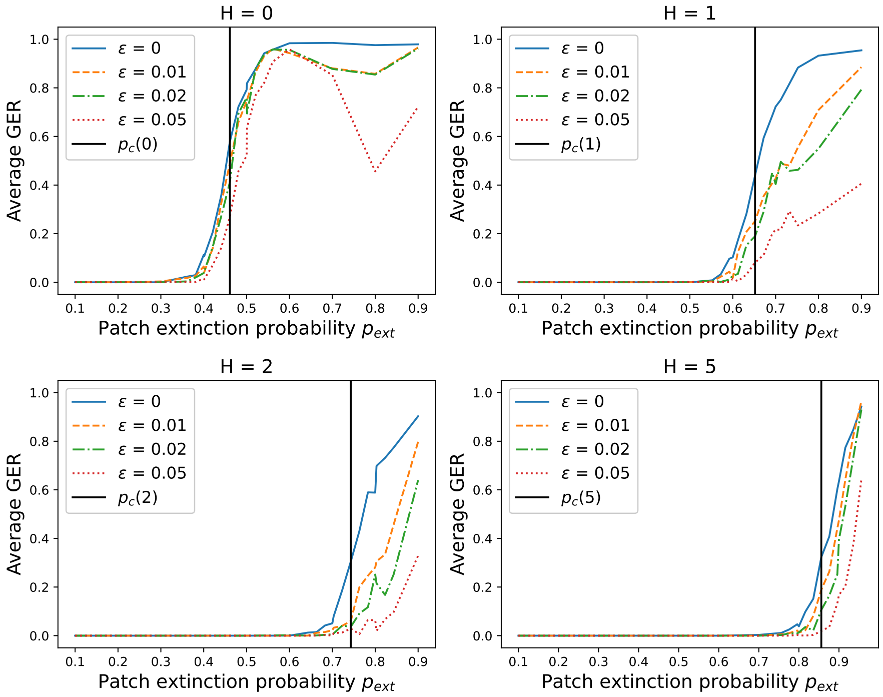

Figure B

$(N, T) = (100, 5)$

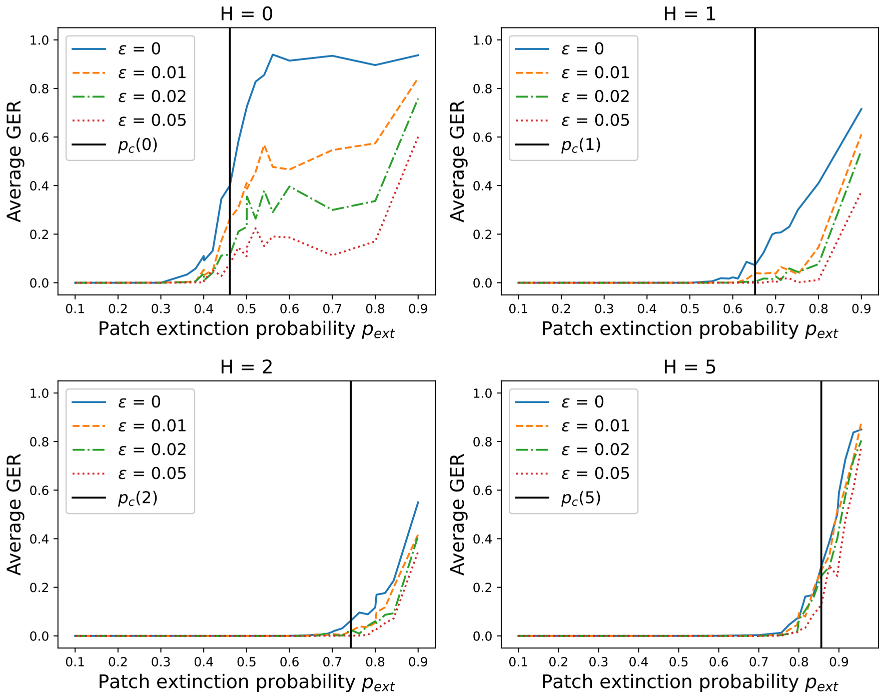

Figure C

$(N, T) = (100, 10)$

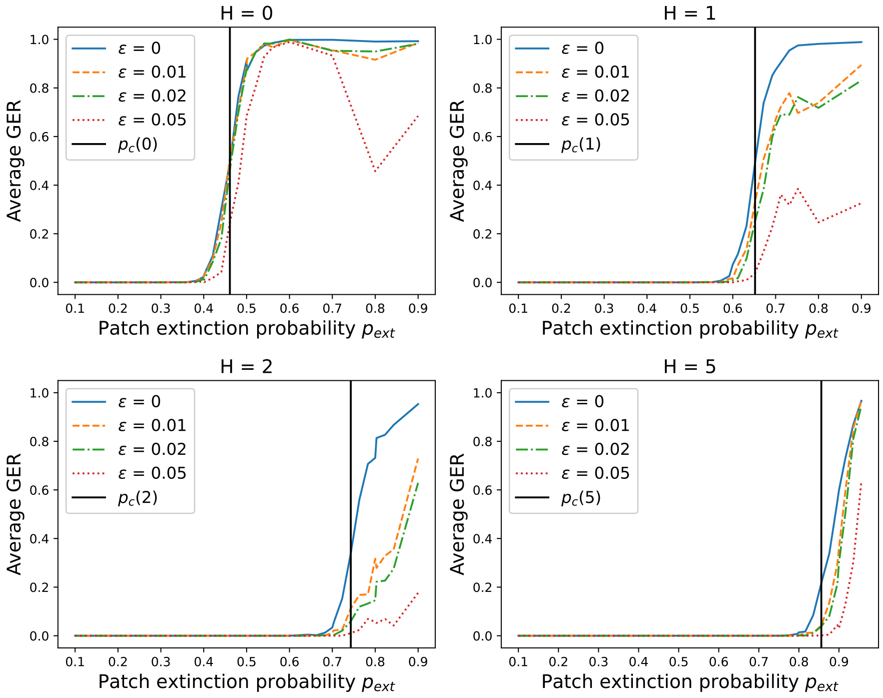

Figure D

2.2 Performances of the MaxGER metric

The following figures show the evolution of the MaxGER metric as a function of the patch extinction probability p_{ext}. For each parameter set listed in Table B, we simulated 30 datasets and performed parameter inference under a noisy BOA process. We then computed the average MaxGER across the 30 simulations. The black vertical line indicates the critical patch extinction probability $p_c(H)$.

$$(N, T) = (50, 5)$$

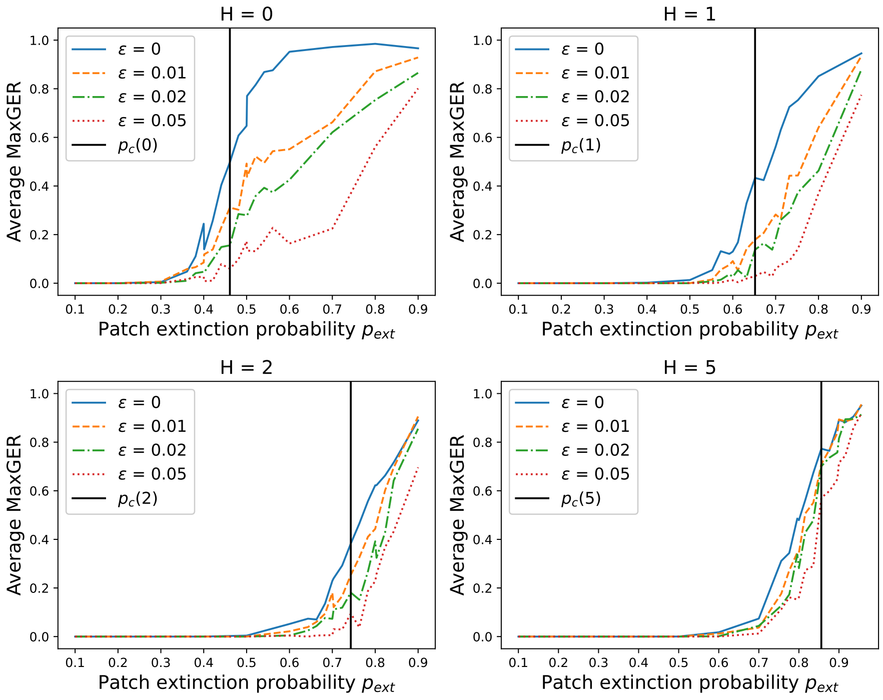

Figure E

$(N, T) = (50, 10)$

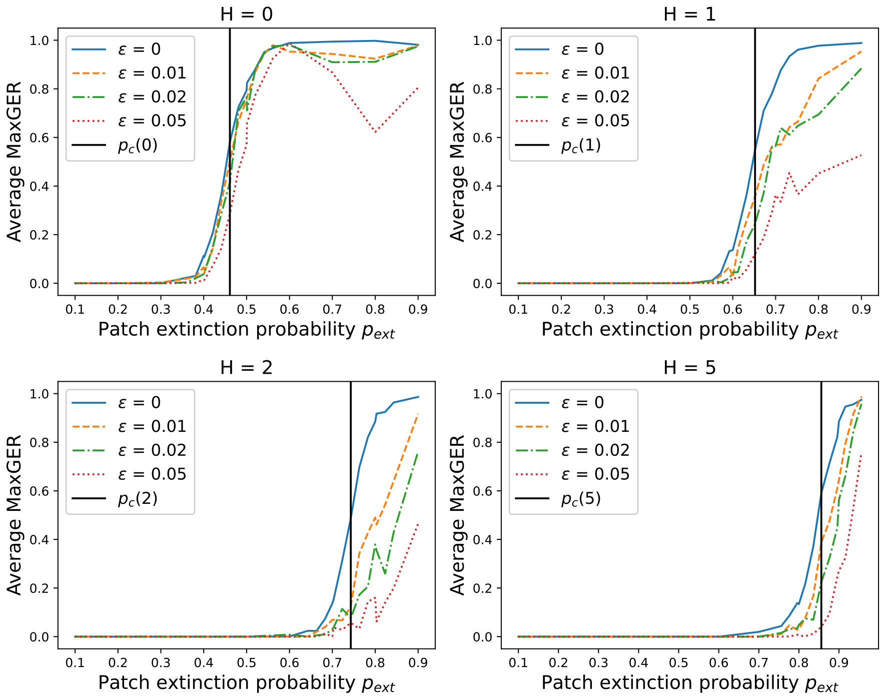

Figure F

$(N, T) = (100, 5)$

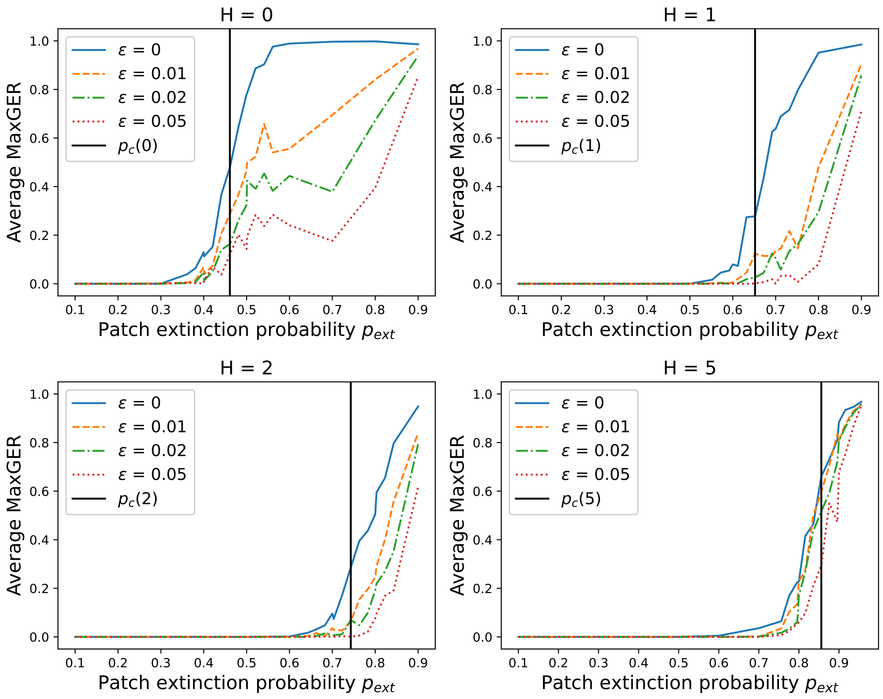

Figure G

$(N, T) = (100, 10)$

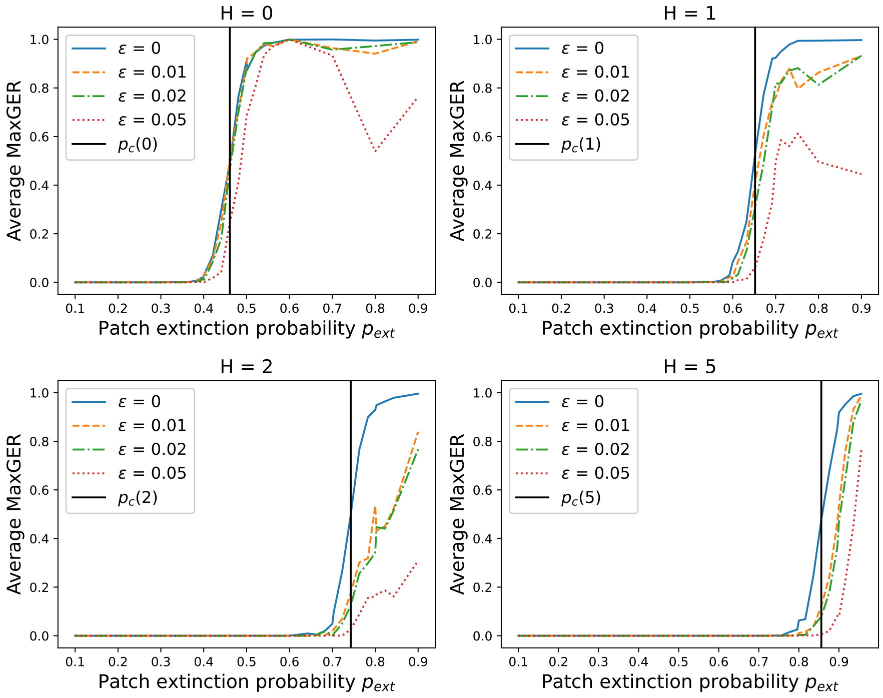

Figure H

In order to assess whether low extinction risks are distinguishable from high extinction risks, we also considered the distribution of the MaxGER metric along its average value. The following figures show the results obtained when $\varepsilon = 0.05$, which can be considered as the worst-case scenario in terms of parameter estimation.

$$(N, T) = (50, 5)$$

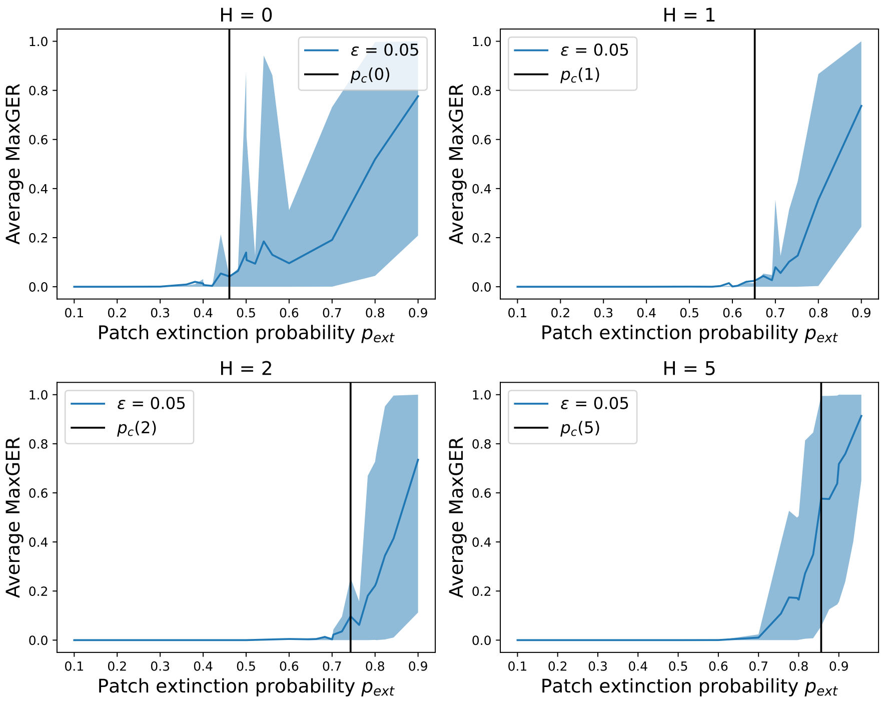

Figure I

$(N, T) = (50, 10)$

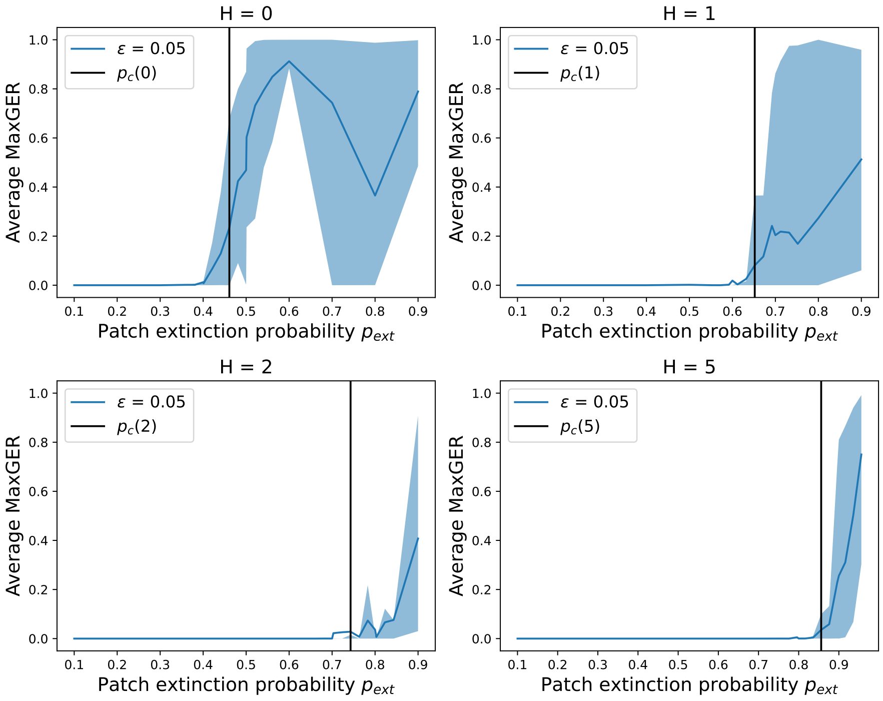

Figure J

$(N, T) = (100, 5)$

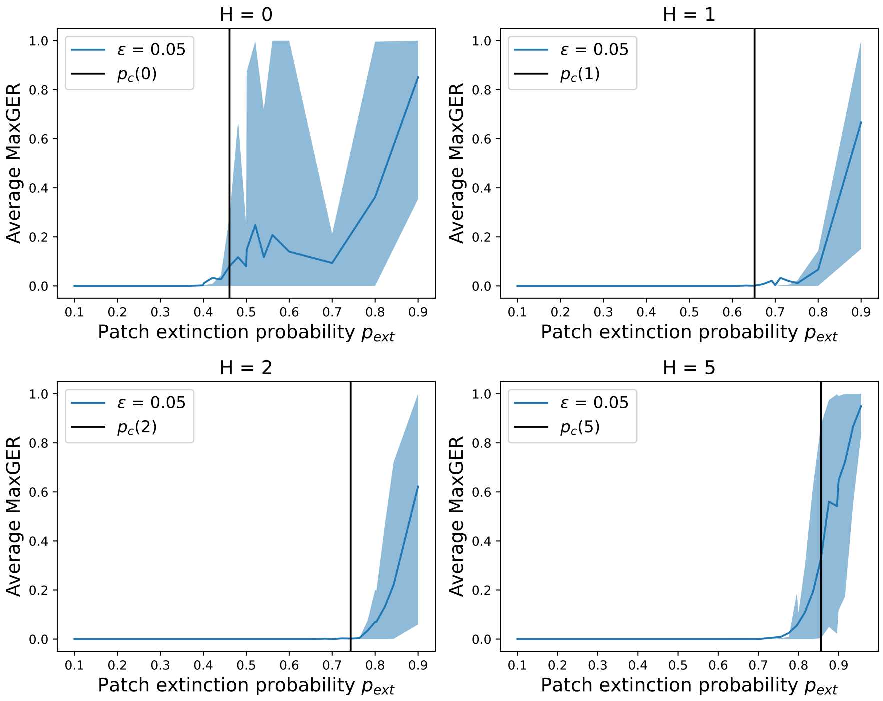

Figure K

$(N, T) = (100, 10)$

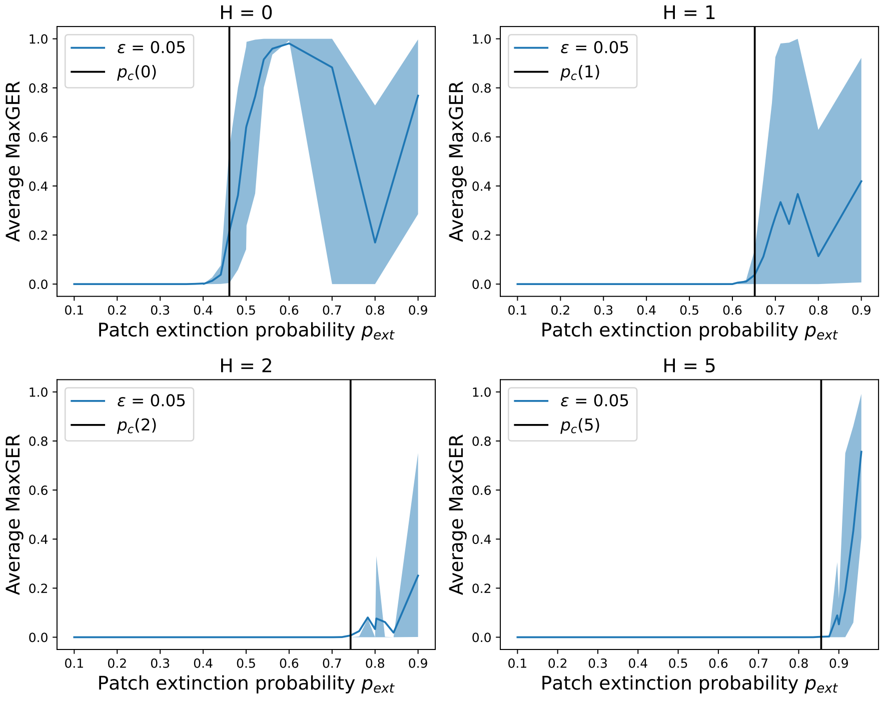

Figure L

2.3 Performances of the MaxGER metric on corrupted datasets

The effect of the introduction of false negatives on the assessment of the MaxGER metric is mostly visible when $H = 0$, for high patch extinction probabilities and when parameter estimation is performed under a BOA process without noise.

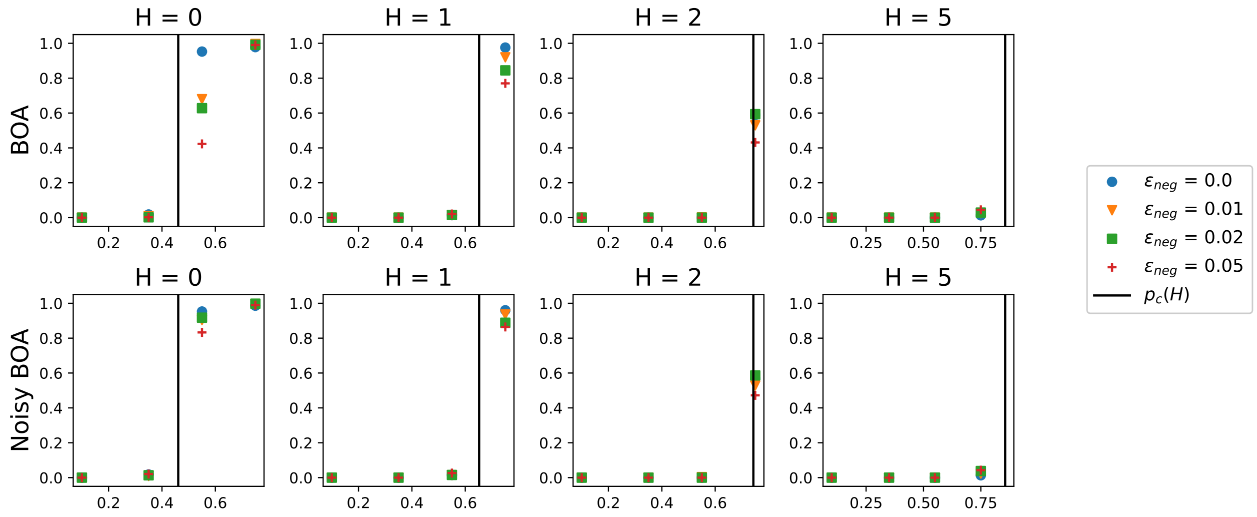

Figure M: Effect of the introduction of false negatives on the assessment of the MaxGER metric, when estimation is performed under a BOA process or a noisy BOA process with $\epsilon_{max} = 0.1$. For each parameter set listed in Table B, we simulated 30 datasets and performed inference under a BOA process or a noisy BOA process with $\epsilon_{max} = 0.1$. The black vertical line indicates the critical patch extinction probability $p_c(H)$.

The introduction of false positives has a very different outcome depending on whether the estimation is performed under a BOA or noisy BOA process. This is particularly visible when $H = 0$ or 1 and for high patch extinction probabilities.

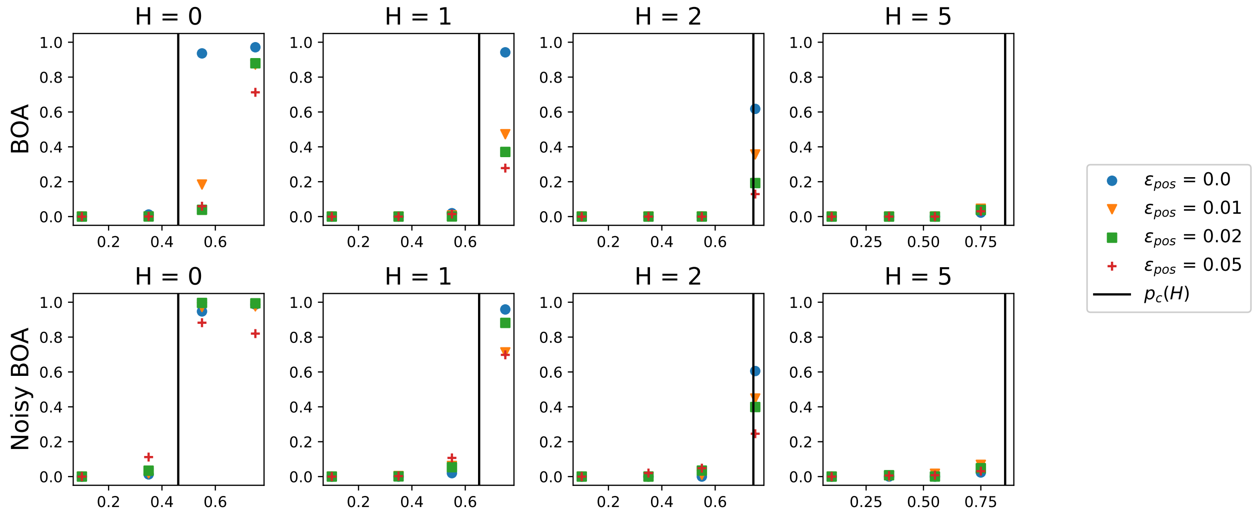

Figure N: Effect of the introduction of false positives on the assessment of the MaxGER metric, when estimation is performed under a BOA process or a noisy BOA process with $\epsilon_{max} = 0.1$. For each parameter set listed in Table B, we simulated 30 datasets and performed inference under a BOA process or a noisy BOA process with $\epsilon_{max} = 0.1$. The black vertical line indicates the critical patch extinction probability $p_c(H)$.

The same observation can be made in the presence of low rates of external colonization.

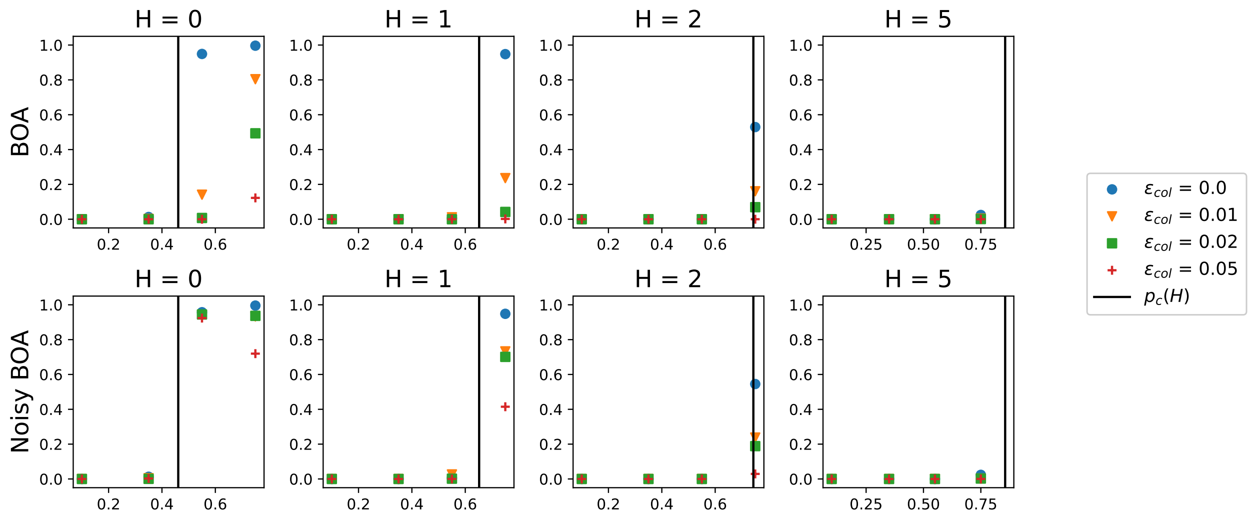

Figure O: Effect of the introduction of external colonization on the assessment of the MaxGER metric, when estimation is performed under a BOA process or a noisy BOA process with $\epsilon_{max} = 0.1$. For each parameter set listed in Table B, we simulated 30 datasets and performed inference under a BOA process or a noisy BOA process with $\epsilon_{max} = 0.1$. The black vertical line indicates the critical patch extinction probability $p_c(H)$.

3 Performances of the estimation of p_{ext} and H

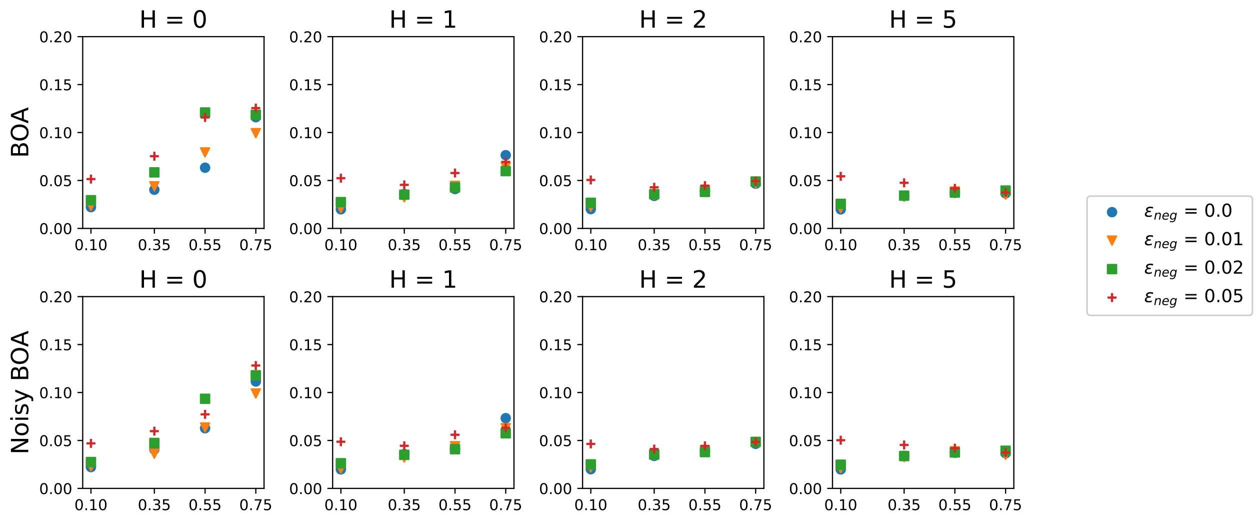

Figure P: Root Mean Square Error (RMSE) on the estimation of p_{ext} in the presence of false negatives, when performing parameter inference under a BOA process or a noisy BOA process with $\epsilon_{max} = 0.1$. For each parameter set listed in Table B, we simulated 30 datasets and performed inference under a BOA process or a noisy BOA process with $\epsilon_{max} = 0.1$.

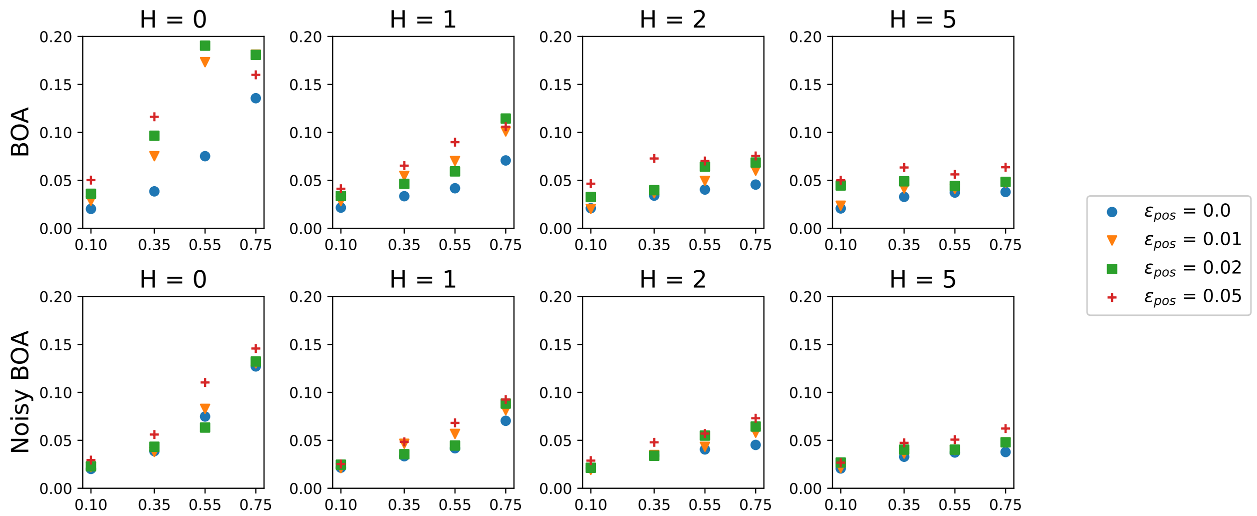

Figure Q: Root Mean Square Error (RMSE) on the estimation of p_{ext} in the presence of false positives, when performing parameter inference under a BOA process or a noisy BOA process with $\epsilon_{max} = 0.1$. For each parameter set listed in Table B, we simulated 30 datasets and performed inference under a BOA process or a noisy BOA process with $\epsilon_{max} = 0.1$.

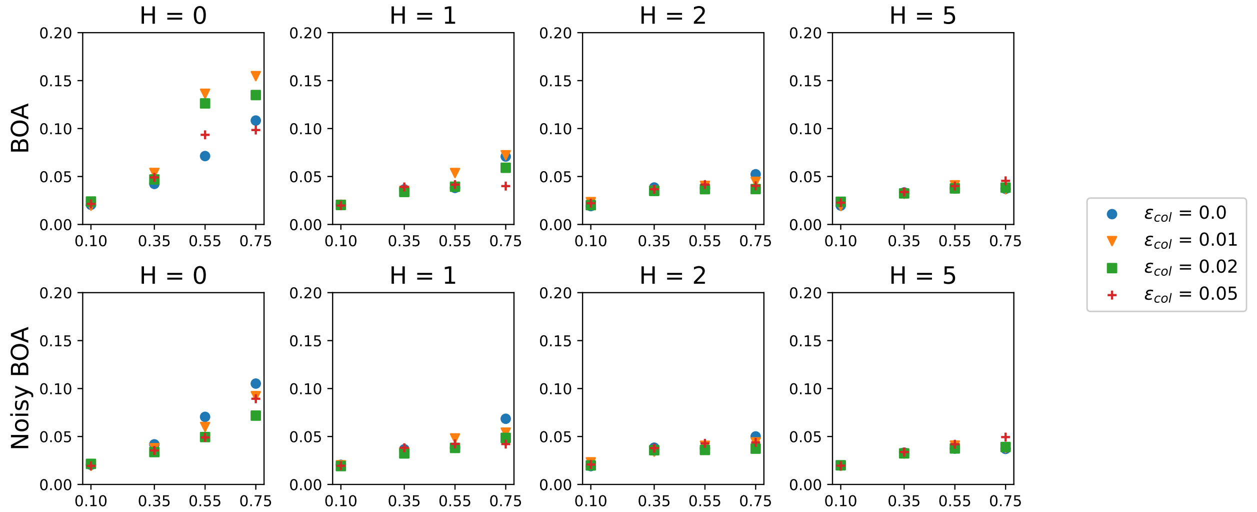

Figure R: Root Mean Square Error (RMSE) on the estimation of p_{ext} in the presence of external colonization, when performing parameter inference under a BOA process or a noisy BOA process with $\epsilon_{max} = 0.1$. For each parameter set listed in Table B, we simulated 30 datasets and performed inference under a BOA process or a noisy BOA process with $\epsilon_{max} = 0.1$.

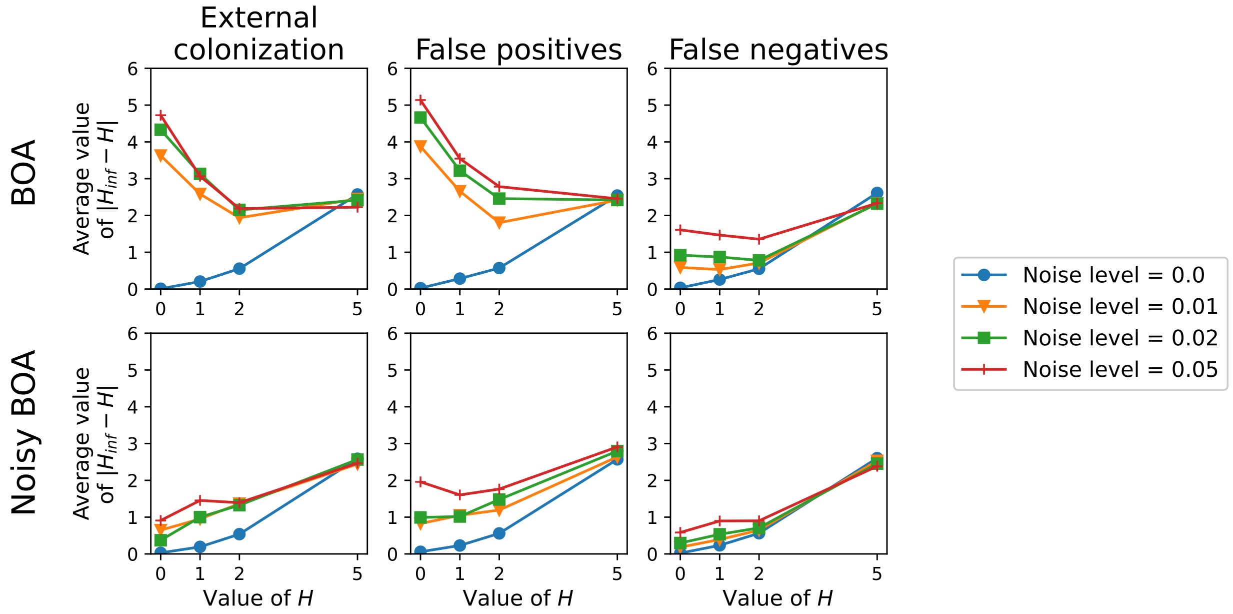

Figure S: Average value of $|H_{inf} - H|$ in the presence of false negatives, false positives or external colonization, when performing parameter inference under a BOA process or a noisy BOA process with $\epsilon_{max} = 0.1$. For each parameter set listed in Table B, we simulated 30 datasets and performed inference under a BOA process or a noisy BOA process with $\epsilon_{max} = 0.1$.

4 Handling multiple streets

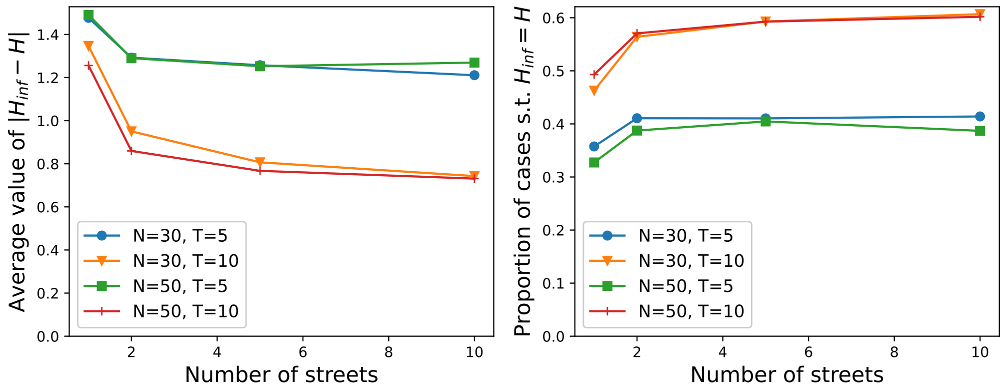

Figure T: Effect of performing the estimation simultaneously on multiple streets on the quality of the estimation of H, when the maximal dormancy duration H and the noise intensity ϵ are constant across streets. The parameter sets used are listed in Table C.
