# Supplementary material for: Assessing the extinction risk of the spontaneous flora in urban tree bases
Source: PLoS Comput Biol. 2024 Jun 27;20(6):e1012191. doi: 10.1371/journal.pcbi.1012191 (PMC11236206; doi:10.1371/journal.pcbi.1012191)
Supplement: Attachment — Submitted filename: LouvetMantouxMachon.pdf [file pcbi.1012191.s004.pdf]

Report on the paper:

Assessing the extinction risk of the spontaneous flora in urban tree bases

by: Apolline Louvet, Clement Mantoux and Nathalie Machon

The authors have adeptly developed and applied statistical methodologies to analyze the Paris yearly floristic inventories dataset, containing information on species presence and absence across over a thousand tree bases. Their approach leverages the BOA process, previously introduced by the first author, to construct a sophisticated Bayesian framework.

Introducing a novel measure, the authors infer the extinction probability of the plants in the study, enabling an ordering of plant species and uncovering intriguing correlations. Notably, the paper delves into the significance of seed production, emphasizing that maintaining a seed bank enables plants to persist over time and mitigates recurrent extinction events triggered by city gardeners cleaning tree bases.

In my assessment, this paper exemplifies the effective integration of a theoretical model for data analysis. The presented statistical framework appears innovative. Moreover, exploring evolution in tree bases strikes a commendable balance between the experimental evolution control and the realism of ecology. Consequently, the development of statistical tools for *Urban Evolution* emerges as a pivotal and promising area of research.

In conclusion, I believe this manuscript constitutes a significant contribution and is well-suited for publication in PLOS Computational Biology following a minor revision.

Comments

- The statistical tools employed in this paper reveal a compelling pattern, highlighting the noteworthy observation that many species with a higher likelihood of survival exhibit a seed bank. I recommend that the authors explicitly acknowledge this as an intriguing observation or delve deeper into its implications. For instance, if the ability to disperse is pivotal for survival in a challenging environment, it would be valuable to explore the strategies employed by species lacking a seed bank to compensate for this deficiency. Could it be that the temporal coordination of actions among gardeners, working closely and at similar times, makes temporary dispersion more efficient than spatial dispersion? In this sense, is the ability to sustain a seed bank particularly important in Urban Evolution? What abilities allow Plantago lanceolata to be so successful in other environments, but not in this one? The authors are encouraged to either clarify the current status of this observation or provide a more in-depth analysis to enrich the discussion.

- What do you mean when you say that your study can be used to design improved management strategies for tree bases? Improve in which sense?

- On page 3, line 24, you make reference to the SPONTANEOUS storage of seeds. This choice of wording might lead readers to expect a discussion on non-spontaneous seeding strategies, which, upon further examination, doesn't seem to be the case. While you may choose to retain the term "spontaneous," consider the option of removing it for clarity.

- In the caption of Figure 2, it would be beneficial to explicitly mention that the curve presented is theoretical. Although this information is clear in the text, some readers may only focus on the

figures. Additionally, the inclusion of some negative values of H in the box appears somewhat distracting.

- Figure 3 requires improvement. The font size in box c is inconsistent, and the captions of the boxes appear twice. Ensuring uniform font size and removing redundant captions, while also centering the remaining captions, would enhance the overall presentation of Figure 3.
