# Supplementary material for: Assessing the extinction risk of the spontaneous flora in urban tree bases
Source: PLoS Comput Biol. 2024 Jun 27;20(6):e1012191. doi: 10.1371/journal.pcbi.1012191 (PMC11236206; doi:10.1371/journal.pcbi.1012191)
Supplement: Attachment — Submitted filename: response_reviewers.pdf [file pcbi.1012191.s005.pdf]

Response to reviewers comments

First, we would like to thank the three reviewers for their insightful and helpful comments and suggestions, which helped us to improve the manuscript. In particular, they allowed us to clarify the exposition of the biological system studied in this article, and we believe the inclusion of these comments and suggestions will help make the article accessible to a wider audience.

Reviewer 1's comments

- *The statistical tools employed in this paper reveal a compelling pattern, highlighting the noteworthy observation that many species with a higher likelihood of survival exhibit a seed bank. I recommend that the authors explicitly acknowledge this as an intriguing observation or delve deeper into its im- plications. For instance, if the ability to disperse is pivotal for survival in a challenging environment, it would be valuable to explore the strategies employed by species lacking a seed bank to compensate for this deficiency. Could it be that the temporal coordination of actions among gardeners, working closely and at similar times, makes temporary dispersion more efficient than spatial dispersion? In this sense, is the ability to sustain a seed bank particularly important in Urban Evolution? What abilities allow Plantago lanceolata to be so successful in other environments, but not in this one? The authors are encouraged to either clarify the current status of this observation or provide a more in-depth analysis to enrich the discussion.*

  Following your comment, we extended the discussion of how our results show that forming a seed bank is essential for survival in urban tree bases (see P.20, L.409-414), and how species that do not form a seed bank do not manage to survive despite lower local extinction risks. We also extended the discussion of the other plant traits improving survival in urban tree bases (see P.21, L.432-440). Note that we did not find any species that could survive in the tree bases in our dataset while not forming a seed bank, which prevented us from exploring the alternative strategies that can be used by a plant species to survive in urban tree bases without forming a seed bank. Instead, we considered the traits that improved survival among species already forming a seed bank, which we believe is also of interest from a biological viewpoint.

  More generally, while it is well-known in population biology that the survived capacity of populations is strongly linked to gene flow, less publications have considered the implications of seed banks for population persistence, and even less so in urban environments. Indeed, the significance of a seed bank for survival in tree bases was overlooked until the work of [Omar et al., 2019]. Therefore, in this article, we chose to focus on the contribution of a seed bank to metapopulation survival.

- *What do you mean when you say that your study can be used to design improved management strategies for tree bases? Improve in which sense?*

  Knowing which species are threatened by which management practices helps to guide more responsible practices for the preservation of biodiversity or of the species the most capable of providing services to urban residents. Following your comment, we have extended the *Author summary* to explicitely state what we mean by "improved management strategies" (see the last line of the *Author summary*).

- *On page 3, line 24, you make reference to the SPONTANEOUS storage of seeds. This choice of wording might lead readers to expect a discussion on non-spontaneous seeding strategies, which, upon further examination, doesn't seem to be the case. While you may choose to retain the term "spontaneous," consider the option of removing it for clarity.*

  We used the word "spontaneous" to distinguish the kind of seed banks we consider from man-made seed banks, but we agree that it might be confusing to the reader. We rewrote this part to remove the word "spontaneous" while still making it clear that we are not referring to man-made seed banks (see P.3, L.29-31).

- *In the caption of Figure 2, it would be beneficial to explicitly mention that the curve presented is theoretical. Although this information is clear in the text, some readers may only focus on the figures. Additionally, the inclusion of some negative values of H in the box appears somewhat distracting.*

  We have modified the legend of Figure 2 (now Figure 3) to make it explicit that the graph represents the evolution of the critical patch extinction probability for the BOA process, rather than for a general metapopulation. Following your suggestion, we also modified the limits of the x-axis and y-axis to exclude negative values for H and $p_c(H)$.

- *Figure 3 requires improvement. The font size in box c is inconsistent, and the captions of the boxes appear twice. Ensuring uniform font size and removing redundant captions, while also centering the remaining captions, would enhance the overall presentation of Figure* 3.

  We modified Figure 3 (now Figure 4) following your recommendations. We also modified Figure A.2, which suffered from similar issues, in the same way.

Reviewer 2's comments

- *As the authors show that their estimates tend to be noisy, it would be nice to show that these second level tests are working as intended I would suggest to use the simulation approach the authors use to assess how different the distributions of extinction risks measures are for different values of the (true) extinction probability (and the other parameters) to assess the power and size of these tests.*

  Following your comment, we performed additional tests to study the distribution of the estimated MaxGER metric along its average value, in order to assess whether the distributions of the MaxGER metric when the extinction risk is low or high are different enough for these cases to be distinguishable. Our results, added to Supporting Information C.2 (see also P.12, L.247-248 in the main document), show that it is generally the case, except potentially when the noise term ε is high and in the absence of a seed bank, where high extinction risks might be underestimated (which turned out not to be an issue in the case of the application to the *Paris* 12 dataset). We chose to only include the results of the additional tests performed for $\varepsilon = 0.05$, as it can be considered as the worst-case scenario among all the ones tested, and already represents 16 additional figures.

  We would also like to mention that our ability to perform additional tests to assess that the second-level tests are working as intended was limited by the fact that we do not have any explicit expression for the true extinction probability at the metapopulation scale, nor for the extinction risk as assessed by the MaxGER metric (which also accounts for uncertainty in parameter estimates, and is thus technically different from the extinction probability).

- *As a possible biological assessment of the validity of their estimates, I encourage the authors to provide a short review of whether dormancy estimates for the species covered are known from germination experiments etc, and, if there are, compare these with their H estimates.*

  In response to your comment, we have now used the LEDA database to check whether the ability to form a seed bank had already been documented for the species considered as part of this study (see P.22, L.458-471). Note that for most species, the maximal dormancy duration was highly dependent on the environment, and often ranged from $H = 0$ to $H > 5$. This, coupled with the fact that we only estimate a lower bound on H rather than the true value of H, prevented us from doing a full comparison of our estimates to data available in the literature. However, we took it as an opportunity to illustrate the need for indirect approaches for the detection of seed bank presence and viability in the environment of interest (see P.22, L.467-471).

- *p.5, l.89-: Please provide an explanation why your model that does not track the number of seeds makes sense biologically (essentially, that you assume the plants provide enough seeds). Is this justified for the species you consider?*

The assumption that is was sufficient to model the presence/absence of seeds rather than their number was made in accordance with previous studies carried out in urban tree bases, along with estimates or measurements of the number of seeds produced by a plant found in the LEDA database, when available. In response to your comment, we extended the first paragraph of the "Analysis of the *Paris* 12 dataset" subsection (see P.12-13, L.253-261) to justify our choice to use a presence/absence-based model to study plant dynamics in urban tree bases. We also separated the definition of the BOA process (a general model, which is well-defined even if it was never biologically relevant) from the discussion of its adequacy to model plant dynamics in urban tree bases, which was moved to another section.

- *p.7, l.124: Describe what is estimated if the true H>10.*

  If $H_{true} > 10$, then the estimator returns what can be interpreted as the relative likelihood of each value of H between 0 and 10, under the assumption that $H_{true} \leq 10$. However, as discussed in the "Estimation method" subsection, unless patch extinction probabilities are extremely high, we cannot expect to differentiate long dormancy durations from medium dormancy durations, even without the upper bound on H, and all relative likelihoods are expected to be similar past a certain value of H. In response to your comment, we extended the discussion in the "Estimation method" subsection (see P.9, L.164-167), in order to explicitly state the implications in terms of the effect of the upper bound on H on the quality of the estimation.

- *p.8, l.133: Can you omit the $\in \mathbb{N}$ here?*

  The equation actually contained a typo, and should read "$H_0 \in \mathbb{N}\backslash\{0, 1\}$". We apologize for the mistake and have now fixed the issue.

- *p.10, Eq. 3: Give a rationale/heuristic of your choice of 0.05 as threshold*

  We have now added above Eq.(3) and at the end of the corresponding paragraph (see P.11, L. 221-223) a quick explanation of our choice of a threshold of 0.05. To summarize, 5% is a standard significance level. Moreover, when implementing the MaxGER metric, we initially also tested several other possible thresholds, and the 0.05 threshold was the one for which the MaxGER metric behaved the most as desired. We agree that there is a slightly arbitrary component in the choice of the threshold, as it is the case for each choice of threshold, and this is part of the reason why we performed an extensive assessment of the performance of the MaxGER metric.

- *p.13, l.258: Please refer to an appropriate table/figure here*

  We now added at the end of the paragraph a reference to where the corresponding figures can be found in the Supporting Information file (see P.15, L.305-307).

Reviewer 3's comments

- *At times, I found myself confused by the organization and motivation of the paper, especially after reading the Abstract and Introduction. The paper starts off talking about "tree bases". I suspect that this is not something that is going to immediately resonate with most readers, especially those of PLOS Computational Biology. Similarly, the notion or importance of corridors in the context of conservation biology and urban ecology could be better developed for readers. It seems to me that the paper is mostly motivated by the development of the extinction risk metrics from presence-absence data, and that perhaps this is what makes the research most relevant to PLOS Computational biology. A major portion of the paper is dedicated to describing these methods. While the plant data set is very interesting, is perhaps the novel quantitative angle the development of the BOA process? I probably would have benefited a bit more from some background on the existing models that are used for estimating extinction risks, and what the challenges or needs are for estimating extinction risk in such a study like this one, which I assume has to wrestle with presence-absence data and some inferred dynamics (seed banks and small-scale dispersal).*

Following your comment and two others in the "minor issues" section, we have added a definition of "tree bases", "spontaneous flora", and a quick explanation of why ecological corridors are of interest in ecology. Moreover, we included an illustration of urban tree bases. While a significant portion of our article is indeed on the development of extinction risk metrics based on presence/absence data, we chose to focus on urban tree bases because in its current form, our framework is only applicable to equidistant patches along a line. As discussed at the end of the article, it can be extended to more general metapopulation models, but this requires some additional results on the dynamics of these models, which were already known for the process considered in the article, but are not currently known for more general processes. Following your comment, we added at the end of the introduction a reference to the discussion of how the estimation framework can be extended to more general cases (see P.5, L.68-69).

We also extended the introduction to *Stochastic Patch Occupancy Models*, which now spans two paragraphs (see P.4-5, L.39-59). In particular, we mentioned their use in conservation biology to study extinction dynamics, and the fact that they did not account for seed bank presence until recently. We believe that this new exposition makes our contribution to the SPOM modelling framework clearer. Note that we chose to focus on models suited to perform inference given the data available to us (i.e., only presence/absence data) and making it possible to study the extinction risk, which generally fall into the SPOM category.

- *Perhaps this doesn't change how the data are analyzed, but "metapopulation" studies tend to focus on a single species, while "metacommunities" study the spatial processes regulating the composition of multispecies assemblages, and often consider processes like dispersal and dormancy on dynamics, including extinction. Is there a reason why the paper is focused on metapopulations and not metacommunities? Is it reasonable to assume that the species in a tree base are not interaction with one another? In other words, are extinction risks not affected by the presence-absence of other species either at the local or regional scale? I think this is an important question — or assumption — that needs to be addressed and justified in some way.*

  The main reason why the paper is focused on metapopulations rather than metacommunities is the lack of SPOM metacommunity models with associated statistical tools, even in the absence of a seed bank. Therefore, we had to consider each plant species as independent from other species, in line with previous studies performed on urban tree bases. We have now added a discussion of the fact that we consider independent metapopulations rather than metacommunities is a limit of our study and is due to the limitations of available modelling frameworks and inference tools, and a call for the development of SPOM metacommunity models with a seed bank component (see P.23, L.486-493).

- *The paper talks about traits. It seems a bit awkward to repeatedly say "biological traits" in a paper that is about plants, as there are no other traits that would be relevant to the study. It is interesting that flowering time, maximum height, and flowering duration are related to extinction risk. The discussion of these traits is somewhat underdeveloped. I was also curious about the associated statistical analyses. Flowering period and flowering duration are treated categorically. What type of correlation was performed? And why were only p-values reported and not correlation coefficients, which would inform reader about the strength of the association? How are these traits related to dispersal or seed banks?*

  Our reason for using the expression "biological traits" what that we initially expected to find correlations with street characteristics, and we wanted to use a terminology differentiating species traits from environment traits. However, it turned out that contrary to previous studies on urban tree bases, we did not find an influence of the street on the quantity of interest as part of this study. Therefore, while there are other traits that might have been relevant to the study, we agree that our repeated use of "biological traits" sounds awkward, and we changed it to "traits", "plant traits" or "species traits".

  Regarding the statistical analyses, the tests performed are described on P.14, L.286-289 and in Table 1. In particular, as the quantities tested were not normally distributed (as we now

mention on P.14, L.287-288 following your comment), we had to use nonparametric statistical tests, which made it less informative or not possible to report correlation coefficients, and we instead chose to include boxplots whenever the correlation was significant in order to visualize the strength of the correlation (see Figure 4).

Following your comment and the one of another reviewer, we extended the discussion of the biological traits related to the extinction risk (see P.21, L.432-440), and improved our explanation of the rationale behind our choice of plant traits to analyse (see P.14, L.289-296). In particular, some traits were not all chosen due to a relation to dispersal or seed banks, but due to a potential effect on survival in urban environments: for instance, plants growing high are more likely to be targeted by gardeners, and we wanted to see whether this was actually visible in our dataset.

- *I am a little confused about the support (i.e., data) for the conclusion that tree bases act as ecological corridors. Which data are used to bolster this inference? Perhaps this could be made clearer?*

  The identification of ecological corridors relies on an interesting property of the BOA process (which was arguably not explicitly stated in the manuscript), which is that survival is equivalent to spatial spreading. We agree that this should be made clearer in the manuscript, and we have now added several mentions to this fact throughout the *Material and Methods* section.

- *There is a lot of data in the Supplementary materials. I did not spend an extended amount of time going through this section, but I did note that it contrasts with the relatively small amount of data presented in the main manuscript, which effectively reports (three) trait correlations (Fig. 3) and description of extinction risks for 21 plant species (Fig. 4).*

  While the Supporting Information file is indeed very long, only the first section of that document contains results regarding plant dynamics in urban tree bases. Moreover, all the data included in that section was also included in a summarized way (not necessarily as part of a figure or table) in the main manuscript:

  - Figure A.2 and Table A.6 report the result of the correlation test between the LER and several traits. These results were summarized in P.18-19, L.367-370. As no significant correlation was identified, we made the choice not to include Figure A.2 in the main manuscript.
  - Table A.3 (resp. Table A.5) reports the complete results of the regression of the MaxGER (resp. the LER) on species and portions of streets. These results were reported in the main manuscript in P.16, L.335-338 (resp. P.18, L.364-366).
  - Table A.4 reports the p-values of the tests whose results are reported in Figure 4 of the main manuscript.
  - Table A.7 reports the values of H_{inf} obtained for each species studied. These results are summarized in the main manuscript in P.19, L.373-376.
  - Figure A.3 reports the results summarized in P.16, L.360-362 of the main manuscript.
  - The remaining figures and tables only give additional background information on the dataset used as part of this study.

  While we agree that the Supporting Information file is arguably hard to read as a stand-alone document, whenever it is referenced in the main manuscript, we give a precise reference to where the corresponding information can be found in the SI file. However, following your comment, we have now added at the beginning of the SI file a quick overview of the organization of the document, and of where the summarized results can be found in the main manuscript.

- *The paper seems to invoke an importance of seed banks, but in the Results section, all we're really told is that 19 out of 21 species had a seed bank. It seems that species without seed banks have higher estimated extinction probabilities? Maybe this point could be better emphasized.*

  In this study, we took into account the potential presence of a seed bank due to its implication for the survival of the metapopulation, as illustrated in Figure 3. While only one of the results

stated was explicitly related to seed banks, all the results involving the extinction risk at the global scale were obtained taking into account the potential presence of a seed bank. Moreover, while species without a seed bank or only able to go dormant for short durations seem to have a higher local extinction risk (as measured by the LER), one drawback of our study is that we could only take into account pairs of species and streets in which the focal species was observed sufficiently often. This means that we tend to exclude from the analysis the pairs for which the patch extinction probabilities are very high, which biases the comparison between species with and without a seed bank. Therefore, we chose not to include this comparison in the article.

Following your comment and the one from another reviewer, we have now extended the discussion of the effect of the seed bank on the extinction risk (see P.20, L.409-414).

- *Maybe the paper should define what tree bases are, and perhaps include a picture as Fig. 1*

  See the answer to your first comment.

- *Abstract: "... some of them being not previously known" vague reference/Abstract: "... approach we introduced" vague reference*

  We modified the corresponding sections of the abstract following your comments.

- *Author summary: "we introduced a new method" vague reference/"Our approach takes into account several factors previously identified... " vague reference/"We applied our approach" vague and hard to follow at this stage of paper.*

  Following your comment, we made these parts clearer. As the author summary is supposed to be accessible to a non-scientist audience, we made the choice not to be more precise than indicating that we introduced a "new estimation framework".

- *Line 28: what is meant by "spontaneous flora"?*

  See the answer to your first comment.

- *Line 55: is this a "working hypothesis" (that will be tested) or is it an assumption?*

  We apologize for the confusion, we have now corrected this part.

- *Line 60 (and throughout): "pair species/portion" is sort of awkward and not well defined.*

  We have now partly reorganized the paragraph from L.84 to L.94 to make it clearer what we mean by "pair species/portion of street", and changed this formulation to "pair of plant species and portion of street".

- *Line 143: what are "fake seed bank characteristic events"?*

  We have now added an explanation of what we mean by "fake seed bank characteristic events", see P.9, L.171-172.

- *Line 173: "that allow to identify" fix grammar*

  Thank you for pointing it out, this has now been corrected.

- *Table 1: I'm not familiar with the dispersal mechanisms described in the "values taken" column*

  We have now added a quick description of each of the dispersal mechanisms listed in Table 1.

- *I think the data is publicly available, but it might be good for them to make accessible the data they used along with code, which I don't see any reference to (e.g., github, zenodo, etc.)*

  We have to admit we do not entirely understand this comment, because as stated in the Data Availability Statement, the data was made available on Zenodo, and the code on Github. We incldued detailed readme files explaining how to rerun the codes, all the codes used as part of the study (including the codes used to generate figures), and the requirements in terms of package versions. We agree that reproducibility of results is important and tried to make the Github repository as user-friendly as possible, and we would welcome any more detailed advice in that sense.
